# Square-Planar vs. Trigonal Bipyramidal Geometry in Pt(II) Complexes Containing Triazole-Based Glucose Ligands as Potential Anticancer Agents

**DOI:** 10.3390/ijms22168704

**Published:** 2021-08-13

**Authors:** Alfonso Annunziata, Davide Liberti, Emiliano Bedini, Maria Elena Cucciolito, Domenico Loreto, Daria Maria Monti, Antonello Merlino, Francesco Ruffo

**Affiliations:** Dipartimento di Scienze Chimiche, Università di Napoli Federico II, Complesso Universitario di Monte S. Angelo, Via Cintia 21, 80126 Napoli, Italy; alfonso.annunziata@unina.it (A.A.); davide.liberti@unina.it (D.L.); emiliano.bedini@unina.it (E.B.); cuccioli@unina.it (M.E.C.); domenico.loreto@unina.it (D.L.); mdmonti@unina.it (D.M.M.); antonello.merlino@unina.it (A.M.)

**Keywords:** platinum(II), square-planar complexes, glycoconjugation, cytotoxic activity, DNA binding

## Abstract

This article describes the synthesis, characterization, and biological activity of novel square-planar cationic platinum(II) complexes containing glucoconjugated triazole ligands and a comparison with the results obtained from the corresponding five-coordinate complexes bearing the same triazole ligands. Stability in solution, reactivity with DNA and small molecules of the new compounds were evaluated by NMR, fluorescence, and UV–vis absorption spectroscopy, together with their cytotoxic action against pairs of immortalized and tumorigenic cell lines. The results show that the square-planar species exhibit greater stability than the corresponding five-coordinate ones. Furthermore, although the square-planar complexes are less cytotoxic than the latter ones, they exhibit a certain selectivity. These results simultaneously demonstrate that overall stability is a fundamental prerequisite for preserving the performance of the agents and that coordinative saturation constitutes a point in favor of their biological action.

## 1. Introduction

Organometallic complexes have been currently considered valid anticancer agents due to their unique features [1,2,3]. Platinum compounds play a prevalent role in metal-based anticancer therapies, with cisplatin, carboplatin, and oxaliplatin used all over the world in clinics despite general low selectivity and drug resistance often limiting their efficacy [4,5]. To date, the efforts of the scientific community have multiplied to prepare increasingly effective agents, aimed at reducing the numerous and unpleasant side effects of cisplatin and its close derivatives [6,7,8,9,10,11,12,13,14,15,16]. One of the most successful strategies is the introduction of bio-active molecules in the coordination environment of the metal, capable of increasing its versatility and selectivity [17,18]. Among these, sugars are an excellent example due to their biocompatibility, the possibility of modulating their chemico-physical properties, and the ability of selective recognition by cancer cells, known as the Warburg effect [19,20,21,22,23,24,25,26,27,28]. In this context, over the last few years, our research group has proposed new organometallic glycoconjugate platinum complexes as innovative anticancer agents [29,30,31,32,33].

**Figure 1:**
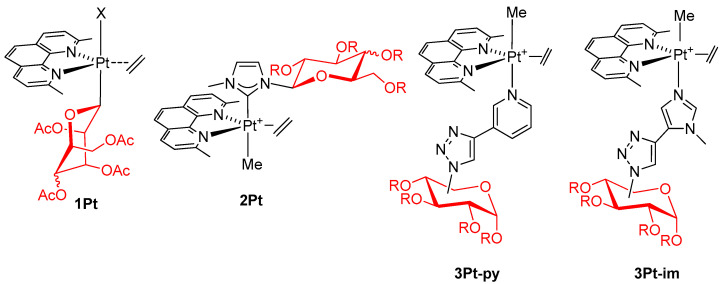
Structure of the five-coordinate platinum(II) complexes **1Pt**, **2Pt**, and **3Pt** (R=H, OAc).

In particular, we intensely explored five-coordinate platinum(II) complexes (Figure 1) characterized by trigonal bipyramidal geometry, with the equatorial plane occupied by the bidentate ligand 2,9-dimethyl-1,10-phenantroline (dmphen) and ethylene, while the sugar fragment occupies one of the axial positions [34]. These species combine glycoconjugation with coordinative saturation, a prerogative that is supposed to increase the stability of the structure and preserve its integrity up to the cellular target. The biological results confirmed the expectations because **1Pt** [30] and **2Pt** [31] showed, in some cases, remarkable activity and selectivity towards pairs of tumor and healthy cell lines, while the entire panel of complexes **3Pt** [29] displayed high activity, although poor selectivity.

**Figure 2:**
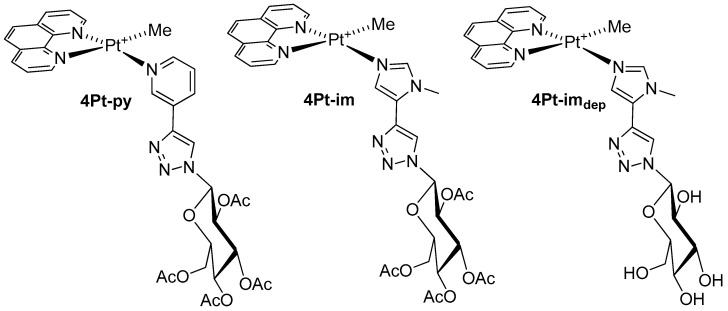
Structure of the four-coordinate platinum(II) complex **4Pt**.

Since the versatility of platinum organometallic chemistry allows us to span between different coordination numbers and geometries, we were stimulated to synthesize new carbohydrate-based platinum agents, **4Pt** (Figure 2), to better understand the effect of the coordination environment on cytotoxicity as well as selectivity:

These compounds share with the five-coordinate **3Pt** agents the oxidation state II of Pt, a positive charge, a glycoconjugate ligand, and a methyl group. On the other hand, they are four-coordinate, and the bidentate nitrogen chelate dmphen is replaced with 1,10-phenanthroline (phen), which does not introduce steric hindrance in the coordination plane and, hence, is more suitable in square-planar environments. It is noteworthy that the planarity of the molecules, combined with the presence of aromatic moieties, phen, and triazo-pyridine/imidazole ligands, makes the family of **4Pt** potential metallo-intercalating agents, capable of intercalating in DNA base pairs by π–π stacking and weak electrostatic interactions [35,36,37,38]. Representative complexes present, respectively, pyridine (**4Pt-py**) and imidazole (**4Pt-im**), decorated with a peracetylated glucose and a ligand in which the sugar is completely deprotected (**4Pt-im_dep_**). To the best of our knowledge, this is a rare example of a homogeneous comparison between two classes of Pt-based anticancer agents sharing an oxidation state and the nature of the ligands in two different molecular geometries [39].

In this paper, we describe the synthesis and spectroscopic characterization of the new compounds, their in-solution stability and reactivity with DNA, and their cytotoxic activity in comparison with **3Pt** analogs.

## 2. Results

### 2.1. Synthesis and Characterization of Complexes of Type 4Pt

**Scheme 1:**
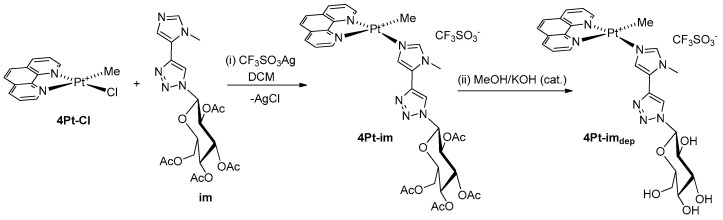
Synthesis of the four-coordinate platinum(II) complex **4Pt-im**.

**Figure 3:**
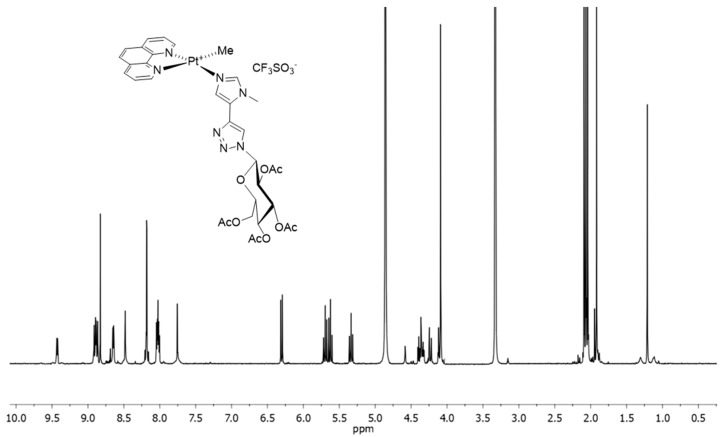
^1^H NMR spectrum of **4Pt-im** in MeOD at 400 MHz and 25 °C.

The sugar ligands **py**, **im**, and **im_dep_** were obtained through a click chemistry reaction between peracetylated 1-azido-β-d-glucose and the appropriate heterocyclic precursor 3-ethynylpiridine or 5-ethynyl-1-methyl-1H-imidazole. The peracetylated platinum(II) complexes were then prepared, starting from the chloro-precursor [PtClMe(phen)], by exchanging halogen in the presence of silver triflate, as exemplified in Scheme 1 for **4Pt-im** (path i):

After filtration of the precipitated silver chloride, the complexes were isolated as yellow powder by reducing the volume of the filtrate and adding diethyl ether. The corresponding deprotected species, **4Pt-im_dep_**, was prepared by treatment of **4Pt-im** in methanol containing a catalytic amount of potassium hydroxide. Attempts to isolate the square-planar dmphen analogs were performed by reacting the chloro–dmphen precursor in the same conditions reported in Scheme 1. Unfortunately, the reaction mixture showed no clear presence of the desired product. The unsuccessful outcome of the synthesis is probably due to the steric hindrance brought into the plane by the methyl groups, which prevents the formation of the square-planar complex. Finally, complex **4Pt-py** was prepared uneventfully with a procedure analogous to the synthesis of **4Pt-im**. The characterization of the products was carried out through mono- and bi-dimensional NMR spectroscopy (Figures S1–S9), which allowed the unequivocal assignment to the entire panel of protons. Figure 3 shows the proton spectrum of **4Pt-im**.

As expected, the two halves of the phen ligand are not equivalent. The signal of the methyl on platinum shifts to higher frequencies (δ 1.2–1.3) than the five-coordinate species (δ 0–0.5) [29,40], and is affected by the coupling with the ^195^Pt nuclei of ca. 70 Hz, typical of square-planar Pt-complexes containing *N*,*N*′-chelating ligands [41]. The sugar proton signals exhibit the characteristic pattern of the ^4^C_1_ glucose chair, with the glucose in the β-configuration. The compounds were unequivocally identified by HRMS (ESI/QTOF), carried out in methanol. In the spectra, it was possible to observe peaks relative to the cationic platinum complex at m/z 869.2225, 866.2106, and 701.1802, attributable to **4Pt-im, 4Pt-py**, and **4Pt-im_dep_**, respectively (Figures S10–S12).

### 2.2. In-Solution Behaviour and Reactivity with Model Nucleophiles

As mentioned before, **3Pt** complexes are highly cytotoxic but poorly selective. Furthermore, similar IC_50_ values were recorded for the entire panel of compounds, regardless of the nature of the sugar ligand [29]. This behavior suggests that the neutral nitrogen ligands in **3Pt** are vulnerable to substitution in a physiological environment, according to the equation (L= **py** or **im**):

**3Pt-L** + solvent = **3Pt-solvent** + L

Some experiments have demonstrated the ease of substitution in coordinating solvents that is plausibly facilitated by the *trans*-effect of the Me ligand [29]. Therefore, the active species likely do not contain the sugar label anymore, and their activities are very similar.

To compare the solution stability of the complexes, ^1^H-NMR and UV–vis spectra over time were recorded for **4Pt** type species dissolved in diverse aqueous (phosphate buffer, PB; pH 7.4): organic solvent mixtures, i.e., 1:1 *v/v* D_2_O:acetone-*d*_6_; 9:1 *v/v* PB:DMSO-*d*_6_; 1:1 *v/v* PB:DMSO-*d*_6_ and DMSO-*d*_6_. These solvents were selected to verify the stability in pseudo-physiological conditions in the presence of solvents with different coordinating properties and concentrations.

**Figure 4:**
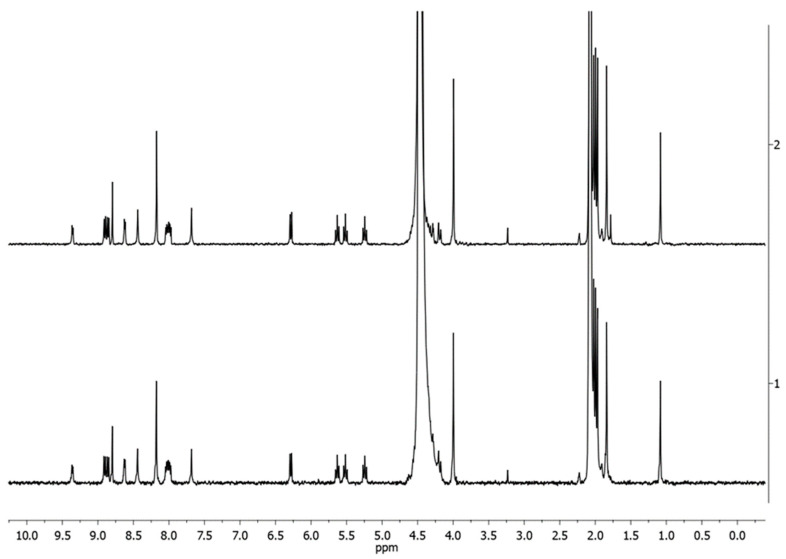
^1^H NMR spectra of **4Pt-im** in 1:1 *v/v* D_2_O:acetone-*d*_6_ over time at 400 MHz, 298K (trace 1 = start, 2 = 72 h).

No appreciable structural variations were observed in the 1:1 *v/v* D_2_O:acetone-*d*_6_ mixture over 3 days. As an example, Figure 4 reports the ^1^H-NMR spectra recorded over time for **4Pt-im**.

**Figure 5:**
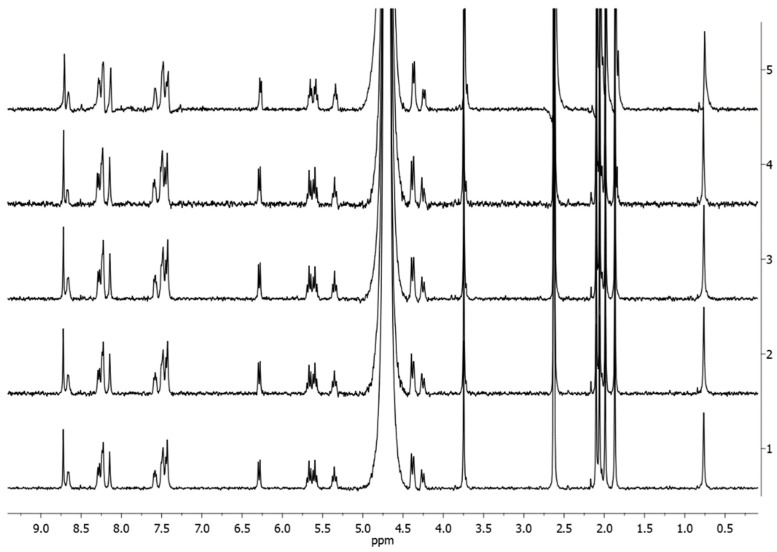
^1^H-NMR spectra of **4Pt-im** in 9:1 *v/v* PB:DMSO-*d*_6_ (1 mM) at 37 °C at different times (1 = start, 2 = 1 h, 3 = 3 h, 4 = 24 h, 5 = 48 h).

Analogous results were obtained in 9:1 *v/v* PB:DMSO-*d*_6_, where only traces of complex **4Pt-dmso-d_6_** were observed after 48 h (Figure 5).

These results demonstrate that coordinating molecules such as water or acetone do not change the nature of the compounds, different from the corresponding five-coordinate **3Pt** complexes that are not stable in these solvents [29].

Upon increasing the amount of DMSO-*d*_6_, type **4Pt** complexes slowly undergo the following exchange:

**4Pt-L** + DMSO-*d*_6_ = **4Pt-DMSO-d_6_** + **L**

**Figure 6:**
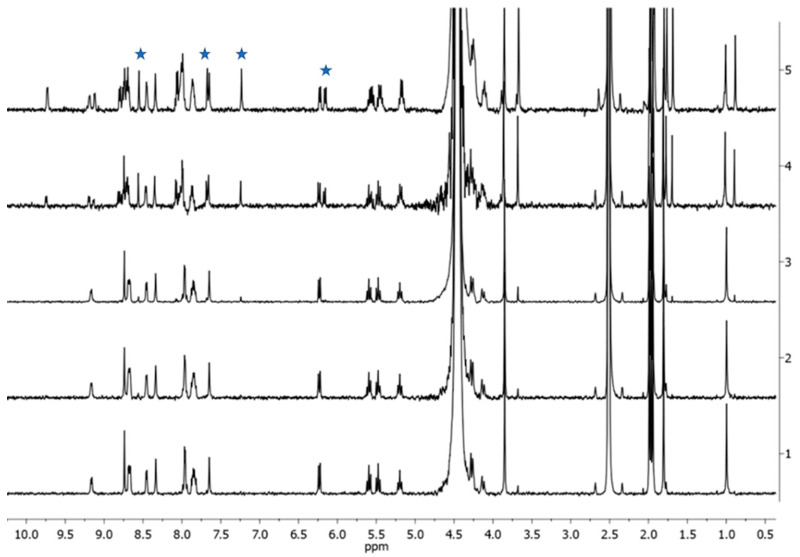
^1^H NMR spectra of **4Pt-im** in 1:1 *v/v* PB:DMSO-*d*_6_ over time at 37 °C (trace 1 = start, 2 = 1 h, 3 = 3 h, 4 = 24 h, 5 = 48 h). Stars indicate the signals of free **im**.

From the spectra recorded in 1:1 *v/v* PB:DMSO-*d*_6_ (Figure 6), it is possible to appreciate the appearance of the signals (δ 6.15, 7.23, 7.68, and 8.55) relating to the free sugar ligand **im**, whose intensity increases over time. After 24 h, an equilibrium is reached. Analog results were collected for **4Pt-py** (Figures S15–S19)**.** The exchange rate was, however, much higher for the analogous five-coordinate species **3Pt-im**, to the point that equilibrium was established in the solution within an hour.

**Figure 7:**
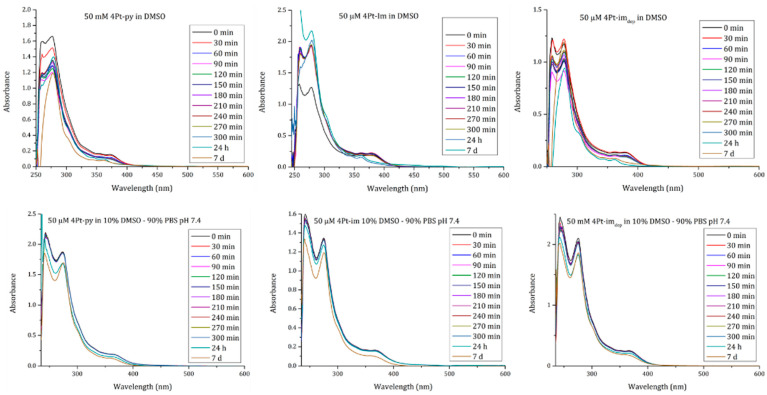
Time course UV–vis spectra of 50 μM **4Pt-im**, **4Pt-im_dep_**, and **4Pt-py** in PBS (pH 7.4):DMSO-*d6* 90:10 (**down**) and pure DMSO (**up**).

These results were confirmed by UV–vis absorption spectra collected in pure DMSO and in 9:1 *v/v* PBS:DMSO-*d_6_* (Figure 7) as a function of time. Indeed, data collected in pure DMSO show significant variations of the spectral profiles, which can be explained by the exchange of a metal ligand with a solvent molecule. On the contrary, spectra collected in the mixed solvent indicate that the compounds remain rather stable in PBS, with a minimal variation of absorbance that can be explained by slight precipitation.

These results demonstrate that four-coordinate complexes preserve their structure more efficiently than the corresponding five-coordinate species in mixed solvents. This behavior can be interpreted by considering the different *trans*-effects experienced by the sugar ligand in the two types of compounds. In five-coordinate complexes, the presence of a methyl destabilizes the Pt–N bond and facilitates the substitution. In the case of square-planar complexes, one phenanthroline nitrogen occupies the *trans* coordination site, and it is therefore reasonable to observe a lower reactivity of the complexes. This result, therefore, encourages the valuation of the biological activity of **4Pt** complexes, with the expectation of a possible recognition action exerted by the sugar portion on the cellular target and a consequent beneficial effect on selectivity.

### 2.3. Cytotoxicity Studies

The biological effect of the Pt-compounds was tested on two human tumor cell lines, MCF-7 (breast cancer cells) and A431 (epidermoid carcinoma cells), and two non-tumorigenic cell lines, H9c2 (rat cardiomyoblast cells), and HaCaT (human keratinocyte cells), using the MTT assay. After 48 h of incubation, a cytotoxic effect was observed in all the analyzed cell lines, as indicated by the IC_50_ values (the amount of drug able to induce 50% cell death) reported in Table 1A. In line with the different stability in solution, the square-planar species of type **4Pt** exhibit different biological activity compared to that of the corresponding five-coordinate compounds of **3Pt**. Indeed, due to their inertness towards the substitution of the sugar ligand, their biological activity can be influenced by the nature of the sugar ligand, whose presence in the complex can affect its internalization or mechanism of action once inside the cell. On the other hand, in the case of the five-coordinate complexes, the loss of the sugar tag plausibly determines their substantial leveling of activities [29]. Indeed, as demonstrated by the selectivity index (Table 1B), the square-planar complexes exhibit a certain degree of selectivity, much higher than that obtained in the case of cisplatin and the corresponding five-coordinate **3Pt** compounds. Although the general cytotoxicity of square-planar species of type **4Pt** is reduced when compared to the coordinatively saturated **3Pt** species, **4Pt-py** showed much higher IC_50_ values on immortalized cells than cisplatin, and a strong decrease in the IC_50_ value, with respect to cisplatin, was observed for A431 cells. The behavior of **4Pt-im** was very similar to that of **4Pt-py**. No toxic activity was observed with **4Pt-im_dep_**, thus confirming the importance of sugar protection, as already observed in other studies [31].

**Figure 8:**
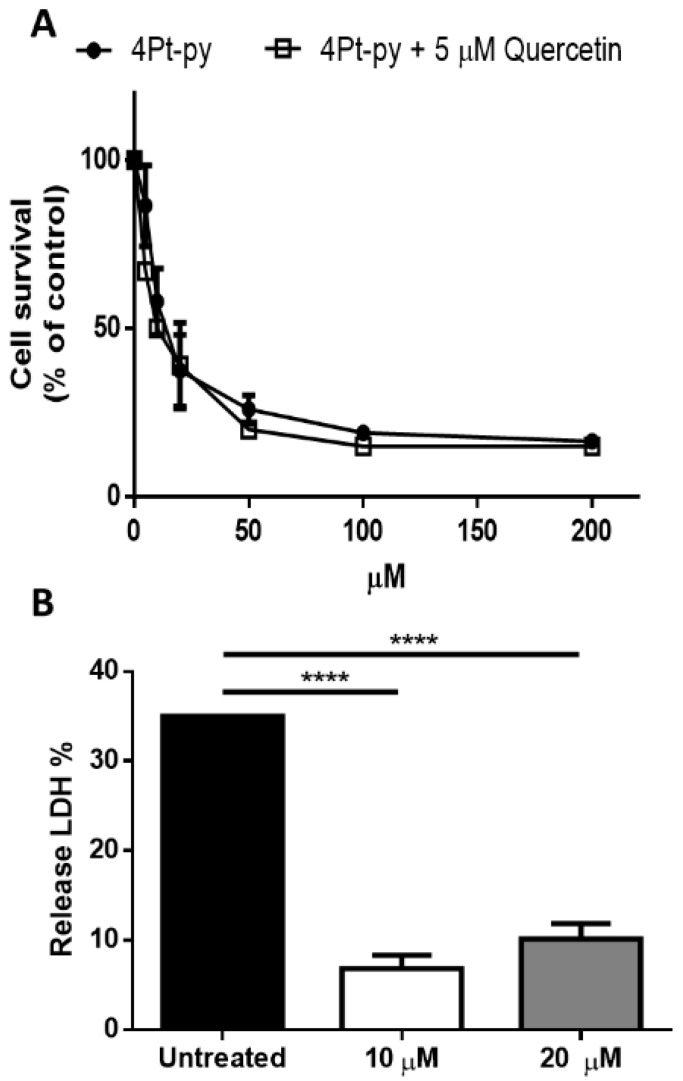
Analysis of the mechanism of action of **4Pt-py**. (**A**) Glucose-transporter-dependent cytotoxicity of **4Pt-py** on A431 cells. Cells were incubated with increasing amounts of **4Pt-py** in the absence (black circles) or presence (empty squares) of 5 μM quercetin. Cell viability was assessed using the MTT assay after 48 h incubation. Values are given as mean ± SD. (**B**) Cells were incubated with increasing amounts of **4Pt-py** for 48 h, and the release of LDH was measured. Data shown are the means ± SD of three independent experiments; **** indicates *p* < 0.001 with respect to untreated cells. The % of LDH release was calculated as described in the Materials and Methods section.

The cell death mechanism induced by **4Pt-py** was analyzed on A431 cells. First, the internalization process of **4Pt-py** was evaluated by studying the involvement of GLUT receptors. In particular, **4Pt-py** internalization and cytotoxicity were tested using quercetin as a glucose transporter inhibitor [24,30,42]. Cells were incubated in the presence or absence of 5 μM quercetin (a non-lethal concentration, data not shown), and a dose–response assay was performed with **4Pt-py**. As shown in Figure 8A, no inhibition of cytotoxicity was observed in the presence of quercetin, thus excluding the involvement of GLUT receptors in the internalization pathway of **4Pt-py**. Necrosis was also excluded as no LDH release was observed after **4Pt-py** cell incubation with an increasing amount of the drug (Figure 8B). The 10 µM concentration used was very similar to the IC_50_ value, and then, a double value (20 µM) was also used to definitely exclude necrosis at very high concentrations.

**Figure 9:**
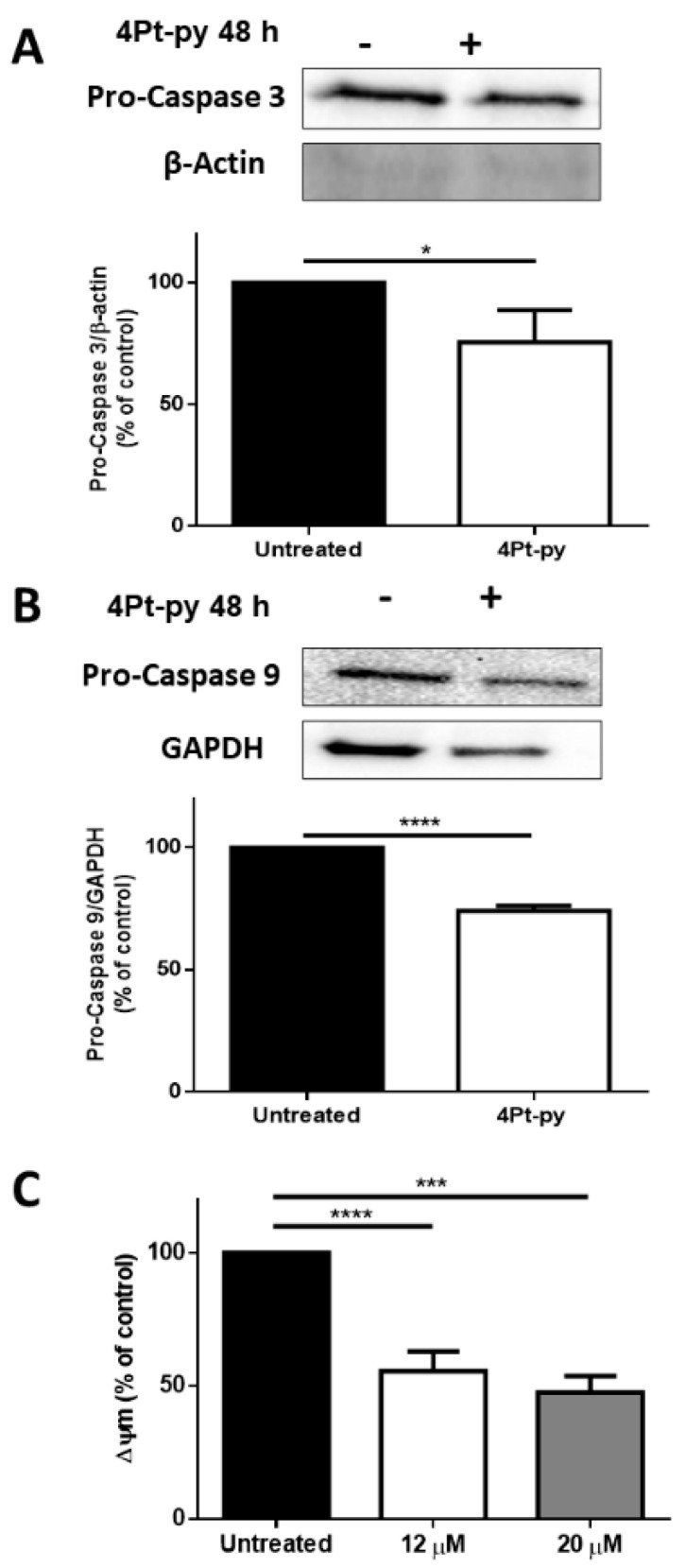
Analysis of 4Pt-py-induced cell death. A431 cells were incubated with 12 μM of **4Pt-py** for 48 h. (**A**,**B**) Analysis by Western blotting of pro-caspase-3 and -9. Β-actin or GAPDH was used as the loading control. The densitometric analysis is reported below each Western blotting. (**C**) Changes in mitochondrial membrane potential (Δψm) after 48 h incubation with an increasing amount of **4Pt-py**. The fluorescence intensity of the probe, related to Δψm, is reported as a percentage of the control (%). Data shown are the means ± SD of three independent experiments; * indicates *p* < 0.05, *** indicates *p* < 0.005, and **** indicates *p* < 0.001 with respect to untreated cells.

The activation of the apoptotic pathway was analyzed by Western blotting and an analysis of the mitochondrial potential as it is well known that mitochondrial outer membrane permeabilization is essential to initiate mitochondrial apoptosis. Cells were incubated for 48 h in the presence of 12 μM **4Pt-py** (the concentration of **4Pt-py** at which the IC_50_ value was obtained), and the activation of caspase-3 and caspase-9 was analyzed. As shown in Figure 9A,B, a small but significant decrease in the signal associated with both pro-caspases was observed. Accordingly, when the mitochondrial potential (Δψm) was measured, a significant decrease in A431 cells was observed at both the IC_50_ value and the doubled amount (20 µM) (Figure 9C).

### 2.4. DNA Binding Properties

Since the main target of Pt-based drugs is DNA, we evaluated the ability of the compounds to interact with nucleobases and double helixes.

**Figure 10:**
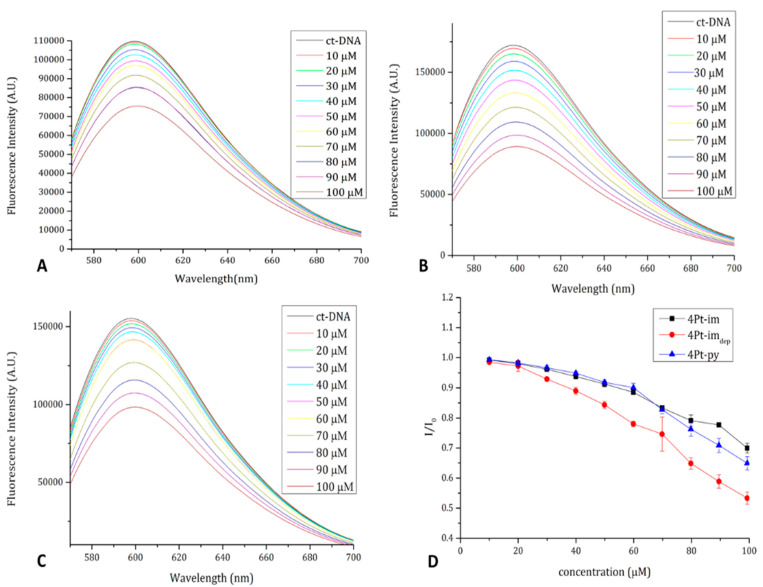
Fluorescence emission spectra of the ct-DNA-EtBr complex upon titration with a solution of **4Pt-im** (**A**), **4Pt-im_dep_** (**B**), and **4Pt-py** (**C**), In panel (**D**), I/Io as a function of compound concentration is reported.

To evaluate the ability of the compounds to interact with double-helix DNA, ethidium bromide (EtBr) displacement fluorescence assays using calf-thymus DNA (ctDNA) were carried out. Attempts to collect circular dichroism spectra of ctDNA in the presence of the compounds failed due to the formation of a yellow precipitate. Results of the fluorescence assay clearly demonstrate that the compounds belonging to the **4Pt** series displace the intercalating agent EtBr from ctDNA (Figure 10). These results indicate that the compounds bind DNA [43].

**Figure 11:**
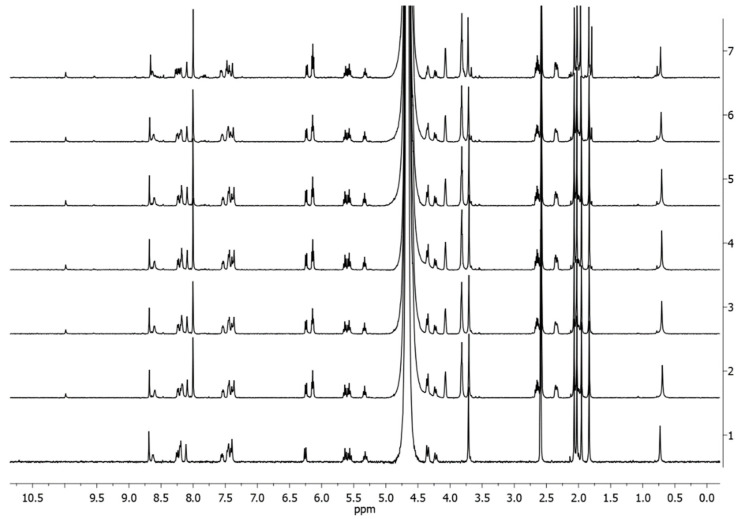
^1^H-NMR spectra over time of **2Pt-im** in 9:1 *v/v* PB:DMSO-*d*_6_ (trace 1) and in the presence of 2.5 eq. of dGMP at 37 °C (trace 2 = start, 3 = 2 h, 4 = 4 h, 5 = 6 h, 6 = 24 h, 7 = 48 h).

To shed light on the interaction between the complexes and DNA, the interaction with the model nucleobase 2′-deoxyguanosine-5′-monophosphate (dGMP) was studied by ^1^H-NMR spectroscopy and ESI mass spectrometry. Indeed, N7 in guanine residues are known to be the main binding sites for platinum complexes. Complexes **4Pt-im** and **4Pt-py** were incubated at 37 °C in 9:1 *v/v* PB:DMSO-*d*_6_ in the presence of 2.5 eq of dGMP, and NMR spectra were recorded over time (Figure 11).

**Figure 12:**
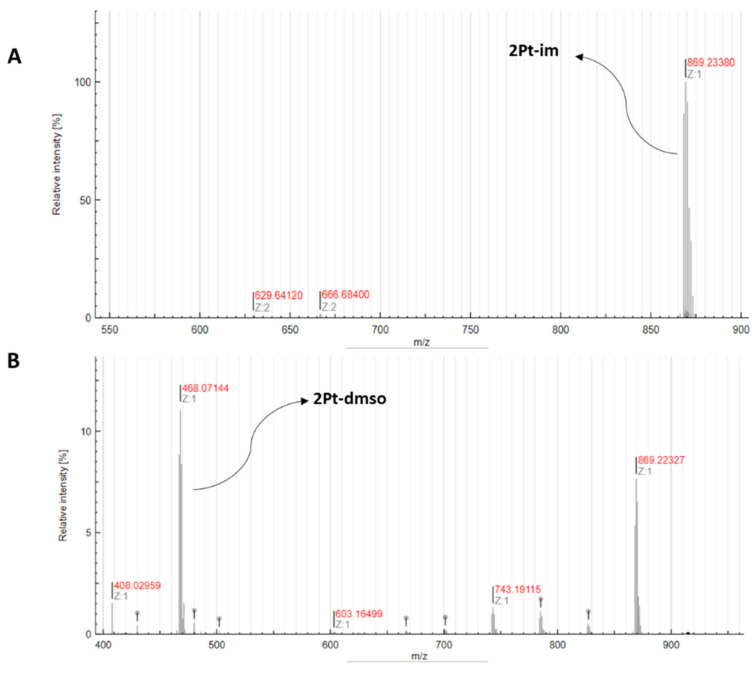
ESI-MS spectrum of **2Pt-im** incubated with dGMP in 9:1 *v/v* H_2_O:DMSO recorded at t = 0 (**A**) and after 48 h at 37 °C (**B**).

After 48 h, no coordination of dGMP to the platinum center was observed, while traces to the complex **4Pt–DMSO** were found. Such results were also confirmed by ESI mass spectrometry experiments performed in 9:1 *v/v* H_2_O:DMSO. The analysis of the reaction mixture at t = 0 (Figure 12A) and after 48 h of incubation at 37 °C (Figure 12B) confirmed the lack of coordination by dGMP and the formation of the **4Pt-DMSO** complex. Analog results were obtained for the **4Pt-py** complex (Figure S19).

Such results, combined with the suppressed ligand exchange in aqueous media (see Section 2.2), may indicate that the family of **4Pt** agents interacts in vitro with DNA through an intercalation mechanism [44]. Indeed, classical Pt(II)-based drugs (e.g., cisplatin and its derivatives), upon the hydrolysis of leaving ligands, can be coordinated by nucleobases, resulting in covalent adducts. The inertness of **4Pt** complexes toward ligand exchange, along with their planarity and the presence of aromatic phenanthroline and triazo-imidazole/pyridine ligands, can favor non-covalent interactions with the double helix [45,46,47,48].

### 2.5. Reactivity with S-Donor Molecules

Due to its softness, platinum forms stable bonds with sulfur-donor nucleophiles, which are ubiquitous in biological environments and play an important role in the fate of platinum-based drugs. Reduced glutathione (GSH) is a tripeptide involved in essential processes in the cell, such as redox balance and detoxification [49]. Platinum complexes promptly react with the thiol group of GSH, forming adducts that are often no longer active. Similarly, the thioether moiety of *L*-methionine is often involved in the sequestration of platinum complexes by proteins [50,51]. Hence, the study of the reactivity of the synthesized complexes with GSH and methionine can help to rationalize and clarify their biological activity. The interaction between compounds **4Pt-im** and **4Pt-py**, incubated at 37 °C in the presence of 2.5 eq. of methionine in 9:1 *v/v* D_2_O (PBS, pH 7.4):DMSO-*d*_6_, was monitored by ^1^H-NMR spectroscopy. Spectra collected over time show no sign of coordination of methionine within 48 h (Figures S20 and S21).

**Figure 13:**
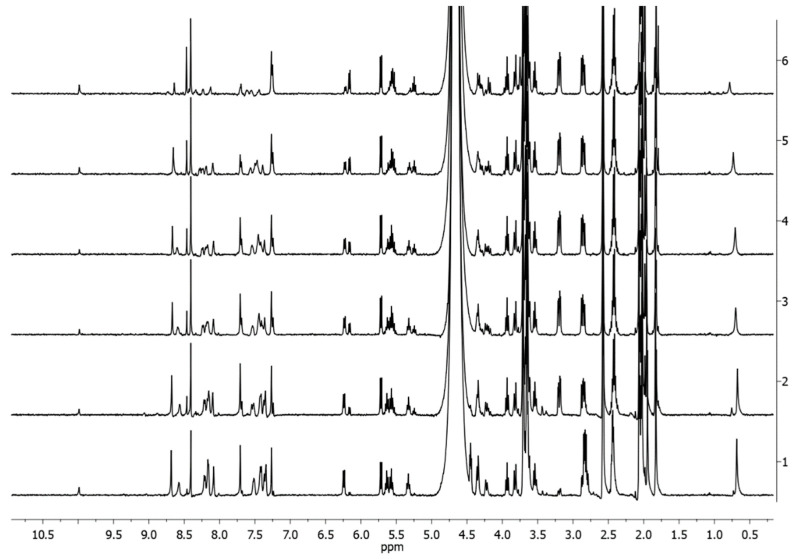
^1^H-NMR spectra of **2Pt-im** incubated with GSH in 9:1 *v/v* PB:DMSO-*d*_6_ (1 mM of Pt, 2.5 eq of GSH) at 37 °C at different times (1 = start, 2 = 2 h, 3 = 4 h, 4 = 6 h, 5 = 24 h, 6 = 48 h).

Conversely, the compounds react with GSH in the same conditions as indicated by ^1^H-NMR and ESI-MS. In the NMR spectra (Figure 13) recorded at different times for **4Pt-im**, it is possible to observe from the point of t = 0 the appearance of new signals in the aromatic and glucose regions.

**Figure 14:**
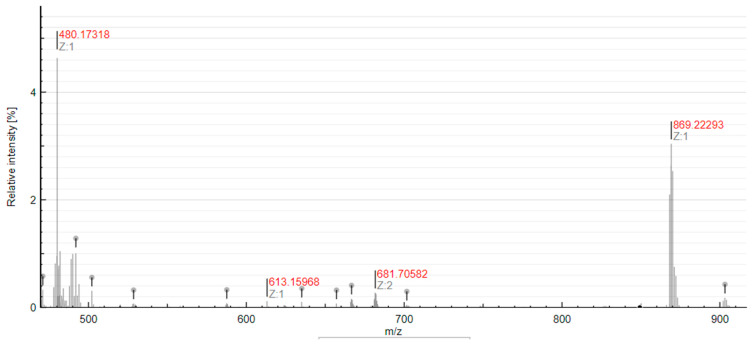
ESI-MS spectrum of **2Pt-im** incubated with GSH in 9:1 *v/v* H_2_O:DMSO, recorded after 48 h at 37 °C.

Such resonances can be attributed to the release of the imidazole ligand and the consequent formation of unidentified species. Such peaks decrease in intensity over time until they almost disappear (in the case of **4Pt-py**, completely; Figure S22), probably because of the precipitation of decomposition products. The reaction mixture analyzed by ESI-MS immediately after the incubation of **4Pt-im** with GSH at 37 °C in 9:1 *v/v* H_2_O:DMSO showed the presence of the intact complex at m/z 869.22. The analysis, repeated after 48 h, revealed the presence of multiple peaks (Figure 14 and Table S1) attributable to several species, including mono- and di-nuclear platinum complexes with one or two deprotonated GSH molecules, probably with bridging sulfur atoms in the di-nuclear one, as previously reported for other phenantroline Pt(II) complexes [51].

## 3. Materials and Methods

Solvents and reagents were purchased from Sigma-Aldrich and used without further purification. NMR spectra were recorded using a 400 Bruker AvanceUltrashielded™ or 500 Varian Inova spectrometer. The chemical shifts are provided in parts per million (ppm, δ), referring to the solvent (^1^H NMR: CHD_2_OD, δ = 3.34 ppm; ^13^C NMR: ^13^CHD_2_OD, δ = 49 ppm). Coupling constants are expressed in Hz. The following abbreviations describe NMR multiplicities: singlet (s), doublet (d), triplet (t), quartet (q), double doublet (dd), broad (br), and multiplet (m). ESI-MS spectra were recorded on a Xevo G2-S QTOF instrument (Waters) in positive ion mode. MS spectra were analyzed using the open-source software *Aom2S* [52].

### 3.1. Synthesis of 4Pt-py and 4Pt-im

An equimolar solution (5.0 mL) of the appropriate ligand in dichloromethane and a suspension of [PtClMe (1,10-phen)] (0.10 g, 0.22 mmol) and CF_3_SO_3_Ag (0.058 g, 0.22 mmol) in dichloromethane (3.0 mL) were stirred at RT for 24 h. After that, the reaction mixture was filtered on Celite^®^, and the filtrate was concentrated under vacuum; diethyl ether was added to afford a yellow microcrystalline solid. The solid was then isolated, washed with diethyl ether, and then vacuum dried (yield: 70–75%).

**4Pt-py** ^1^H NMR (400 MHz, CD_3_OD) ^1^H NMR (400 MHz, MeOD) δ 9.55 (d, *J*_H2Py-H4Py_ *=* 1.9 Hz, *J_Pt_*= 46 Hz, 1H, H2Py), 9.47 (dd, 1H, H2, or H9 phen), 9.03 (dd, *J_H6Py-H5Py_* = 5.6 Hz, *J_H6Py-H4Py_* = 1.3 Hz, 1H, H6Py), 9.01 (s, 1H, H-Triazole), 8.97 (dd, 1H, H4, or H7 phen), 8.93 (dd, 1H, H7, or H4 phen), 8.64 (ddd, *J*_H4Py-H5Py_ = 8 Hz, 1H, H4Py), 8.48 (dd, 1H, H9, or H2 phen), 8.26 (ABq, 2H, H5, and H6 phen), 8.09 (dd, 1H, H3, or H8 phen), 8.04 (dd, 1H, H8, or H3 phen), 7.83 (ddd, 1H, H5Py), 6.29 (d, *J_H1-H2_* = 8.8 Hz, 1H, H1-glu), 5.66 (t, *J_H2-H3_* = 9.3 Hz, 1H, H2-glu), 5.61 (t, *J_H3-H4_* = 9.3 Hz, 1H, H3-glu), 5.32 (t, *J_H4-H5_* = 9.4 Hz, 1H, H4-glu), 4.39 (dd, *J_H6-H6′_* = 12 Hz, *J_H5-H6_* = 5.0 Hz, 1H, H6-glu), 4.33 (ddd, *J_H5-H6′_* = 1.7 Hz, 1H, H5-glu), 4.23 (dd, 1H, H6′-glu), 2.09 (s, 3H, OAc), 2.07 (s, 3H, OAc), 2.04 (s, 3H, OAc), 1.90 (s, 3H, OAc), 1.32 (s, 3H, *J_Pt_* = 81 Hz).

^13^C NMR (101 MHz, MeOD) δ 170.8, 170.0, 169.8, 169.2, 152.7, 150.0 (x2), 148.5, 147.5, 145.0, 142.7, 139.4, 138.7, 135.4, 131.0, 130.7, 130.3, 127.7, 127.6, 127.5, 126.6, 125.61, 121.9, 120.4 (q, *J_C-F_* = 319 Hz), 85.4, 74.7, 72.6, 70.8, 67.8, 61.6, 19.2, 19.1 (x2), 18.7, −12.1.

HRMS (ESI/QTOF) m/z 866.2106, [**4Pt-py**—CF_3_SO_3_^−^].

**4Pt-im** ^1^H NMR (400 MHz, CD_3_OD) δ ^1^H NMR (400 MHz, MeOD) δ 9.44 (dd, J_Pt_ = 53 Hz, 1H, H2, or H9 phen), 8.92 (dd, 1H, H4, or H7 phen), 8.88 (dd, 1H, H7, or H4 phen), 8.83 (s, 1H, H-Triazole), 8.66 (dd, 1H, H9, or H2 phen), 8.49 (br, 1H, H-Imidazole), 8.18 (ABq, 2H, H5, and H6 phen), 8.04 (dd, 1H, H3, or H8 phen), 8.02 (dd, 1H, H8, and H3 phen), 7.76 (d, 1H, H-imidazole), 6.30 (d, J _H1-H2_ = 9.4 Hz, 1H, H1-glu), 5.70 (t, 1H, J _H2-H3_ = 9.4 Hz, H2-glu), 5.62 (t, J _H3-H4_ = 9.4 Hz, 1H, H3-glu), 5.34 (t, J_H4-H5_ = 9.4 Hz, 1H, H4-glu), 4.42–4.32 (m, 2H, H5-glu, and H6-glu), 4.23 (dd, J_H5-H6′_ = 11.8 Hz, J_H6-H6′_ = 1.3 Hz, 1H, H6′-glu), 4.09 (s, 3H, Me-Imidazole), 2.09 (s, 3H, OAc), 2.06 (s, 3H, OAc), 2.04 (s, 3H, OAc), 1.92 (s, 3H, OAc), 1.21 (s, 3H, J_Pt_ = 74 Hz).

^13^C NMR (101 MHz, MeOD) δ 170.8, 170.0, 169.8, 169.2, 149.6, 148.7, 147.7, 145.2, 141.6, 138.9, 138.3, 136.3, 131.0, 130.6, 128.5, 127.6 (x2), 127.4, 126.4, 125.6, 122.3, 120.4 (q, *J_C-F_* = 320 Hz) 85.5, 74.7, 72.5, 70.8, 67.8, 61.6, 33.6, 19.1 (x3), 18.7, −14.0.

HRMS (ESI/QTOF), m/z 869.2225, [**4Pt-im**—CF_3_SO_3_^−^].

### 3.2. Synthesis of 4Pt-im_dep_

Complex **4Pt-im** (0.065 g, 0.064 mmol) was treated in 5.0 mL of methanol containing 5% mol of KOH. After 1 h of stirring, the complex was crystallized by slow addition of diethyl ether to the reaction mixture (yield 90%). 

^1^H NMR (400 MHz, CD_3_OD) δ ^1^H NMR (400 MHz, MeOD) δ 9.46 (d, *J_Pt_* = 49 Hz, 1H, H2, or H9 phen), 8.94 (d, 1H, H4, or H7 phen), 8.90 (d, 1H, H7, or H4 phen), 8.71 (s, 1H, 1H, H-Triazole), 8.68 (dd, 1H, H9, or H2 phen), 8.49 (s, 1H, 1H, H-Imidazole), 8.22 (ABq, 2H, H5, and H6 phen), 8.05 (dd, 1H, H3, or H8 phen), 8.03 (dd, 1H, H8, or H3 phen), 7.75 (s, 1H, H-Triazole), 5.76 (d, *J_H1-H2_* = 9.2 Hz, 1H, H1-glu), 4.09 (s, 3H, Me-Imidazole), 4.01 (t, *J_H2-H3_* = 9.1 Hz, 1H, H2-glu), 3.95 (dd, *J_H6-H6′_*= 11.6 Hz, *J_H6-H5_* = 1.4 Hz, 1H, H6-glu), 3.79 (dd, *J_H6′-H5_* = 5.3 Hz, 1H, H6′-glu), 3.70 3.50 (m, 2H, H3-glu, and H4-glu), 1.26 (s, 3H, *J*_Pt_ = 76 Hz).

^13^C NMR (101 MHz, MeOD) δ 149.8, 148.8, 147.4, 145.2, 141.5, 139.0, 138.1, 135.8, 131.0, 130.7, 128.6, 127.6, 127.4, 126.4, 125.9, 125.6, 122.5, 120.5 (*J_C-F_* = 317 Hz), 88.6, 79.9, 77.0, 72.42, 69.9, 60.8, 33.2, −13.7.

HRMS (ESI/QTOF) m/z 701.1802, [**4Pt-im_dep_**—CF_3_SO_3_^−^].

### 3.3. UV–Vis Absorption Spectroscopy

UV–vis absorption spectra of the compounds as a function of time were collected in PBS (pH 7.4):DMSO-*d6* 90:10 and pure DMSO using a Jasco V-650 UV–vis spectrophotometer. Spectra were collected at room temperature using a compound concentration of 50 μM and 1 cm path length cuvettes. The other experimental settings were: 240–450 nm wavelength range, 400 nm/min scanning speed, 2.0 nm bandwidth, and 1.0 nm data pitch.

### 3.4. Cell Culture and the MTT Test

A431, H9c2, and MCF7 cells were purchased from American Type Culture Collection (ATCC). HaCaT cells were from Innoprot (Derio, Spain). Cells were cultured in DMEM (Sigma-Aldrich, St. Louis, MO, USA), supplemented with 10% fetal bovine serum (HyClone, Thermo Scientific, Logan, UT, USA) and antibiotics in a 5% CO_2_ humidified atmosphere at 37 °C. The growth medium of H9c2 cells was implemented with 2 mM L-glutamine and 2 mM sodium pyruvate. Cells were seeded in 96-well plates at a density of 2.5 × 10^3^ cells per well. Different drugs were added at increasing concentrations 24 h after seeding for dose-dependent assays. For the GLUT1-inhibitor-mediated cytotoxicity assay, 24 h after seeding, the cells were treated with 0.5–200 μM **4Pt-py** in the presence or absence of 5 μM quercetin. Quercetin alone was used as the control. After 48 h incubation, cell viability was assessed by MTT assay, as described in a previous study [30]. Control experiments were performed by either growing cells in the absence of the compound or by supplementing the cell cultures with identical volumes of buffer (water:acetone, 1:1 *v/v*). Each value is the mean of three independent experiments, each with three determinations. Significance was determined by Student’s *t*-test.

### 3.5. LDH Release

The occurrence of necrosis was determined by measuring the release of lactate dehydrogenase (LDH) in the culture medium, as described by Sucha et al. [53]. The LDH content of the medium from untreated cells was referred to as a spontaneous release, whereas the LDH total cellular content was determined upon cell lysis. The percentage of LDH release was calculated as:

(1) $$\mathrm{LDH}\;\mathrm{release}\;\left(\%\right)\;=\;[\left(\mathrm{experimental}-\mathrm{spontaneus}\;\mathrm{release}\right)/\left(\mathrm{total}\;\mathrm{content}-\mathrm{spontaneous}\;\mathrm{release}\right)]\times 100$$

Each value is the mean of three independent experiments, each with three determinations. Significance was determined by Student’s *t*-test.

### 3.6. Western Blot Analyses

A431 cells were plated at a density of 3 × 10^5^ cells cm^−2^ in a complete medium for 24 h and then treated for 48 h with 12 µM **4Pt-py**. At the end of the incubation, both untreated and treated cells were analyzed, as described previously for Western blot analyses [30]. Upon the determination of total protein concentration in the supernatant by the Bradford assay, samples were analyzed by SDS-PAGE and Western blot using specific antibodies directed towards procaspase-3 or -9 (Cell Signal Technology, Danvers, MA, USA). β-actin (Sigma-Aldrich) or GAPDH (Thermo Fisher, Rockford, IL, USA) was used as the loading control. Each value is the mean of three independent experiments, each with three determinations. Significance was determined by Student’s *t*-test.

### 3.7. Analysis of Mitochondrial Membrane Potential

Mitochondrial membrane potential (Δψm) was measured as we described [31]. Cells were plated at a density of 2 × 10^4^ cells per well, and, after 24 h, cells were incubated for 48 h with 12–20 µM **4Pt-py**. At the end of the treatment, the cells were incubated with 200 nM of the cationic lipophilic dye tetramethylrhodamine ethyl ester (TMRE) for 20 min at 37 °C. Then, the cells were gently washed with 0.2% BSA in PBS three times, and the fluorescence was measured in a microplate reader with peak λ(ex)/λ(em) = 549/575 nm. Each value is the mean of three independent experiments, each with three determinations. Significance was determined by Student’s *t*-test.

### 3.8. Ethidium Bromide Displacement Fluorescence Assay

Fluorescence spectra were collected on a HORIBA Fluoromax-4 spectrofluorometer at 25 °C using 1 cm path length cuvettes. ctDNA was incubated with ethidium bromide (EtBr) in a 1:50 molar ratio for 30 min at room temperature. Then, the complex was diluted in 10 mM ammonium acetate buffer at pH 7.5 up to a ctDNA final concentration of 200 μM. The ctDNA–EtBr complex was then titrated with a **4Pt** solution (final concentration from 10 to 100 µM, stock concentration 15 mM). Fluorescence emission spectra were recorded at excitation at 545 nm after an equilibration time of 5 min following each addition of the Pt complex.

### 3.9. ^1^H-NMR and ESI-MS In-Solution Studies

The appropriate **4Pt** complex (5 mg) was dissolved in 0.5 mL of DMSO-*d_6_* or acetone-d_6_. Calculated volumes of solution were diluted to 600 µL with the appropriate volumes of PB in D_2_O (25 mM, pH 7.4) and/or DMSO-*d_6_* to provide a 1 mM solution of the complex with the appropriate *v/v* ratio of solvents. Samples were incubated at 37 °C, and NMR spectra were recorded at different times.

To study the reactivity with dGMP, L-methionine, and GSH, stock solutions (10 mM) in PB of each nucleophile were prepared and the correct volume was added to a freshly prepared solution of the complexes in 9:1 *v/v* PB:DMSO-*d*_6_ to afford a final concentration of 1 mM of the Pt complex and 2.5 eq. of the nucleophile.

Samples for ESI-MS analysis were prepared as described for NMR samples, using DMSO and H_2_O instead of deuterated solvents. Prior to analysis, an aliquot of the reaction mixture was withdrawn and diluted with water to get 0.1 mM of Pt-complex solutions.

## 4. Conclusions

This work is part of broad research aimed at studying the biological activity of platinum(II) complexes containing glycoconjugated neutral ligands. The expertise of this team in favoring four- or five-coordination complexes through small structural modifications of the ligands allowed a homogeneous comparison between the activity of analogous cationic complexes in the two different coordination environments. In line with expectations, the two classes of compounds revealed profound differences, starting with the higher stability of the square-planar species with respect to the substitution of the sugar ligand. This aspect determines that their biological activity depends on the nature of the latter, whereas, in the case of five-coordinate complexes, the loss of the sugar tag plausibly provokes the substantial leveling of activities. This also implies that only the square-planar complexes exhibit a certain degree of selectivity, although with reduced cytotoxicity. The ensemble of results, also accompanied by the demonstration that the complexes interact with DNA, adds a piece to the knowledge of the properties of antitumor agents based on platinum(II), highlighting how the optimization of their performance requires the careful choice of ligands in the appropriate coordination geometry.

## Figures and Tables

**Table 1:** (**A**) IC_50_ values (μM) obtained for Pt-derived drugs on immortalized and cancer cells after 48 h incubation. (**B**) Selectivity index, indicated by the ratio between the IC_50_ values of immortalized cells and cancer cells.

**A. MTT IC_50_ 48 h (** **µM)**				
**Cell Line**	**HaCaT**	**A431**	**H9c2**	**MCF7**
**4Pt-py**	66 ± 11	12 ± 2	22 ± 10	35 ± 4
**4Pt-im**	27 ± 7	16 ± 8	53 ± 11	43 ± 7
**4Pt-im_dep_**	>200	>200	>200	>200
**3Pt-py^29^**	0.80 ± 0.14	1.10 ± 0.14	0.35 ± 0.07	0.88 ± 0.11
**3Pt-im^29^**	1.03 ± 0.25	1.10 ± 0.01	0.38 ± 0.04	0.63 ± 0.11
**3Pt-im_dep_^29^**	0.58 ± 0.04	1.00 ± 0.14	0.35 ± 0.07	0.60 ± 0.09
**Cisplatin**	6.6 ± 0.3^31^	39 ± 12^31^	8 ± 2.1	18 ± 1.6
**B.** $Selectivity\;Index=IC_{50}^{immortalized\;cells}/IC_{50}^{cancer\;cells}$				
**Cell Line**	**HaCaT/A431**	**HaCaT/MCF7**	**H9c2/ A431**	**H9c2/ MCF7**
**4Pt-py**	5.5	1.88	1.83	0.63
**4Pt-im**	1.69	0.63	3.3	1.23
**4Pt-im_dep_**	N.A.	N.A.	N.A.	N.A.
**3Pt-py^29^**	0.72	0.89	0.32	0.39
**3Pt-im^29^**	0.91	1.59	0.34	0.6
**3Pt-im_dep_^29^**	0.58	0.96	0.35	0.58
**Cisplatin**	0.17	0.82	0.2	0.44

## Supplementary Materials

The following are available online at https://www.mdpi.com/article/10.3390/ijms22168704/s1.
