# Supplementary material for: Square-Planar vs. Trigonal Bipyramidal Geometry in Pt(II) Complexes Containing Triazole-Based Glucose Ligands as Potential Anticancer Agents
Source: Int J Mol Sci. 2021 Aug 13;22(16):8704. doi: 10.3390/ijms22168704 (PMC8395886; doi:10.3390/ijms22168704)
Supplement: Supplementary file 1 [file ijms-22-08704-s001.zip › ijms-1342307-supplementary.pdf]

Square-planar *vs.* trigonal bipyramidal geometry in Pt(II) complexes containing triazole-based glucose ligands as potential anticancer agents

Alfonso Annunziata [1], Davide Liberti [1], Emiliano Bedini [1], Maria Elena Cucciolito [1], Domenico Loreto [1], Daria Maria Monti [1], Antonello Merlino [1] and Francesco Ruffo [1,*]

[1] Dipartimento di Scienze Chimiche, Università di Napoli Federico II, Complesso Universitario di Monte S. Angelo, via Cintia 21, 80126 Napoli (Italy)
* Correspondence: ruffo@unina.it

Content

Figure S1. ^{1}H-NMR spectrum of **4Pt-im** in MeOD at 25 °C

Figure S2. COSY NMR spectrum of **4Pt-im** in MeOD at 25 °C

Figure S3. ^{13}C NMR spectrum of **4Pt-im** in MeOD at 25 °C

Figure S4. ^{1}H-NMR spectrum of **4Pt-py** in MeOD at 25 °C

Figure S5. COSY NMR spectrum of **4Pt-py** in MeOD at 25 °C

Figure S6. ^{13}C NMR spectrum of **4Pt-py** in MeOD at 25 °C

Figure S7. ^{1}H-NMR spectrum of **4Pt-im$_{dep}$** in MeOD at 25 °C

Figure S8. COSY NMR spectrum of 4Pt-im$_{dep}$ in MeOD at 25 °C

Figure S9. ^{13}C NMR spectrum of **4Pt-im$_{dep}$** in MeOD at 25 °C

Figure S10. ESI-MS spectrum of **4Pt-im** in MeOH

Figure S11. ESI-MS spectrum of **4Pt-py** in MeOH

Figure S12. ESI-MS spectrum of **4Pt-im$_{dep}$** in MeOH

Figure S13. ^{1}H-NMR spectra over time of **4Pt-im** in DMSO at 37 °C

Figure S14. ^{1}H-NMR spectra over time of **4Pt-im** in 1:1 v/v D_2O : acetone-d_6 (1 mM) at 37 °C

Figure S15. ^{1}H-NMR spectra over time of **4Pt-py** in DMSO at 37 °C

Figure S16. ^{1}H-NMR spectra over time of **4Pt-py** in 9:1 v/v PB : DMSO-d_6 at 37 °C

Figure S17. ^{1}H-NMR spectra over time of **4Pt-py** in 1:1 v/v PB : DMSO-d_6 at 37 °C.

Figure S18. ^{1}H-NMR spectra over time of **4Pt-py** in 1:1 v/v D_2O : acetone-d_6 (1 mM) at 37 °C (1 = start, 2 =72 h)

Figure S19. ^{1}H-NMR spectra over time of **4Pt-py** incubated with dGMP in 9:1 v/v PB : DMSO-d_6 at 37 °C

Figure S20. ^{1}H-NMR spectra over time of **4Pt-im** incubated with L-methionine in 9:1 v/v PB : DMSO-d_6 at 37 °C

Figure S21. ^{1}H-NMR spectra over time of **4Pt-py** incubated with L-methionine in 9:1 v/v PB : DMSO-d_6 at 37 °C

Figure S22. ^{1}H-NMR spectra over time of **4Pt-py** incubated with GSH in 9:1 v/v PB : DMSO-d_6 at 37 °C

Figure S23. ESI-MS spectrum of **2Pt-im** incubated with GSH in 9:1 v/v H_2O : DMSO recorded after the dissolution.

Table S1. List of peaks found in the ESI-MS spectrum of the reaction mixture between **4Pt-im** and GSH after 48 h of at 37 °C.

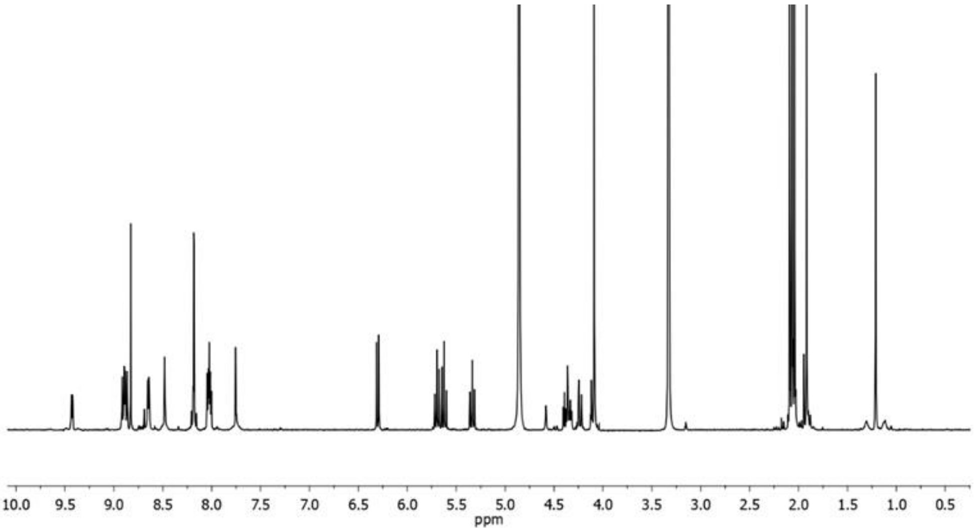

Figure S1. ^{1}H-NMR spectrum of **4Pt-im** in MeOD at 25 °C

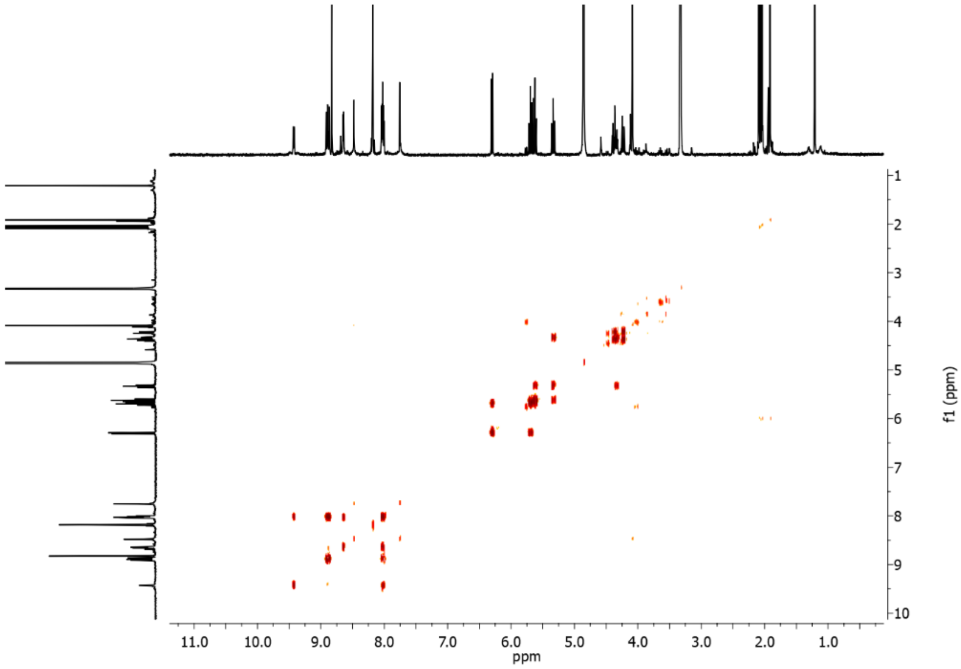

Figure S2. COSY NMR spectrum of **4Pt-im** in MeOD at 25 °C

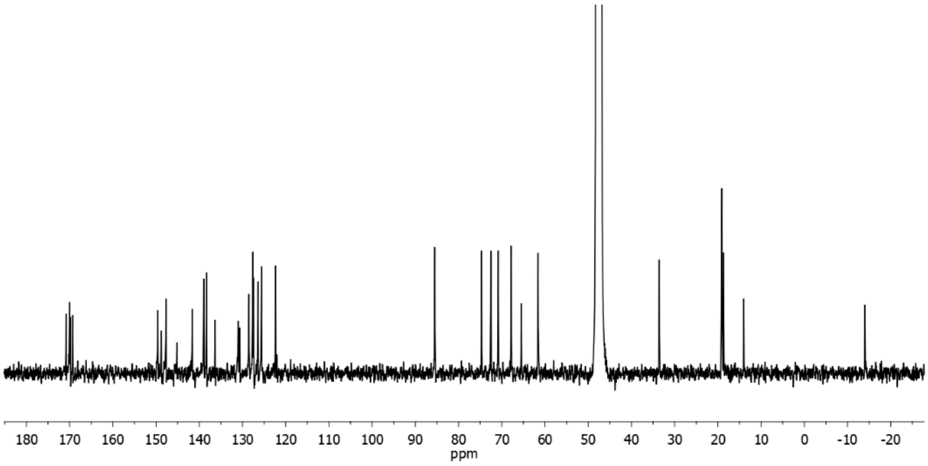

Figure S3. ^{13}C NMR spectrum of **4Pt-im** in MeOD at 25 °C

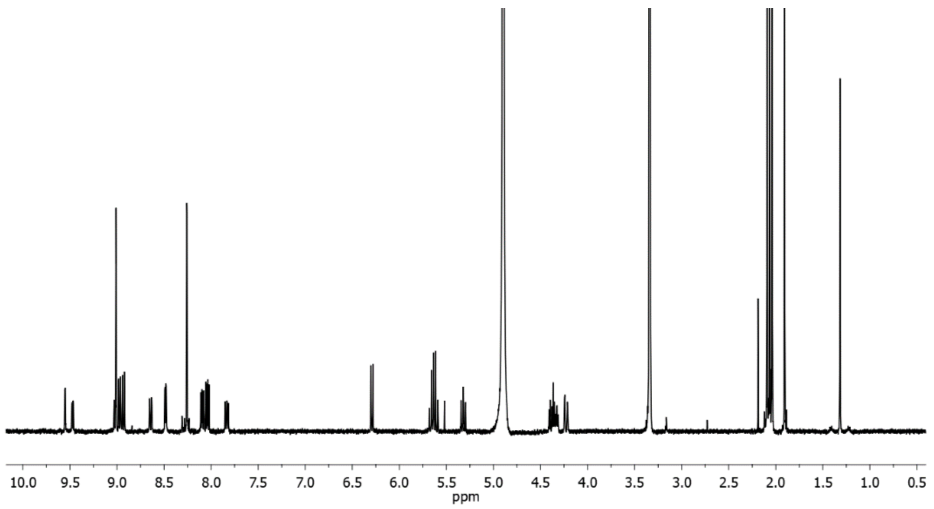

Figure S4. ^{1}H-NMR spectrum of **4Pt-py** in MeOD at 25 °C

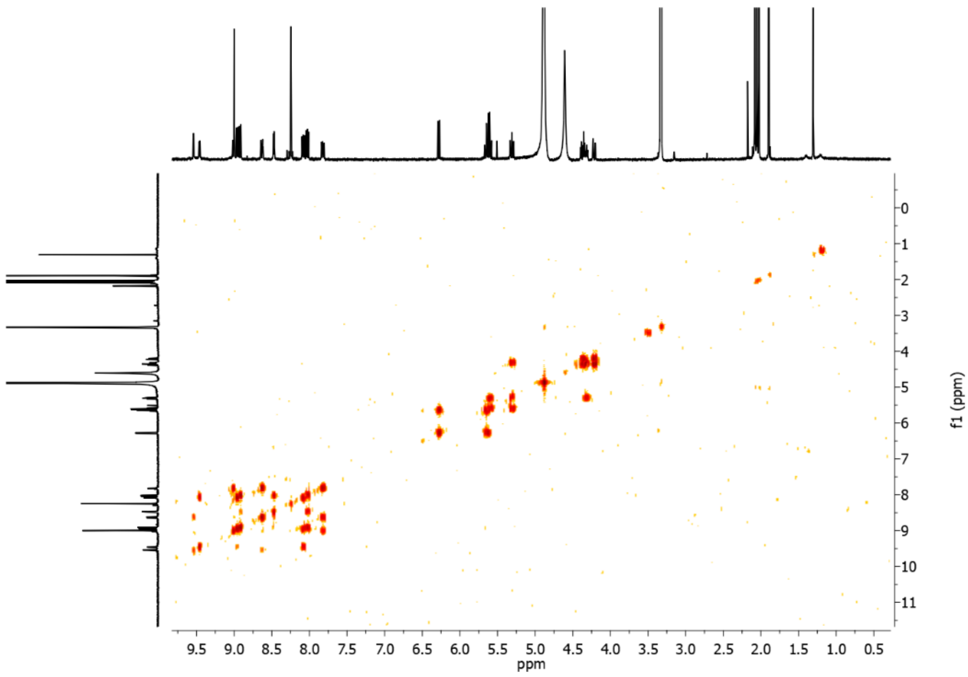

Figure S5. COSY NMR spectrum of **4Pt-py** in MeOD at 25 °C

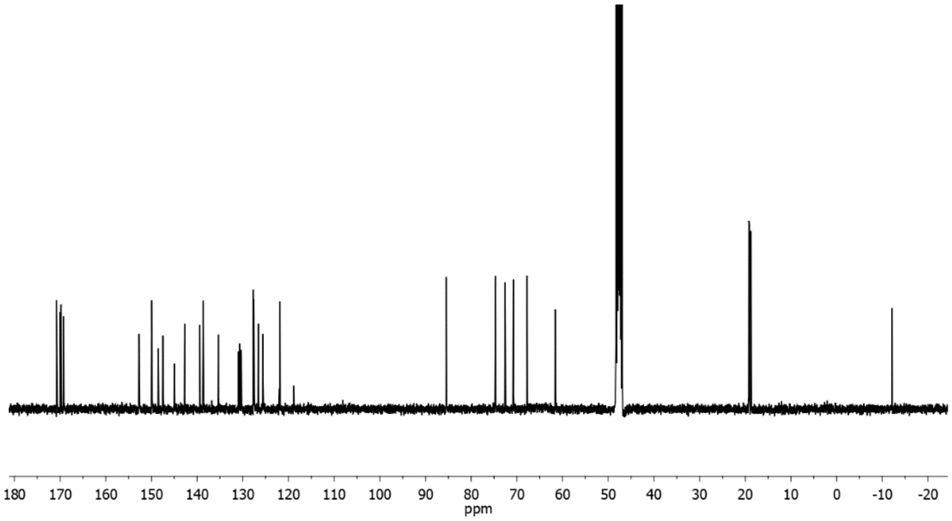

Figure S6. ^{13}C NMR spectrum of **4Pt-py** in MeOD at 25 °C

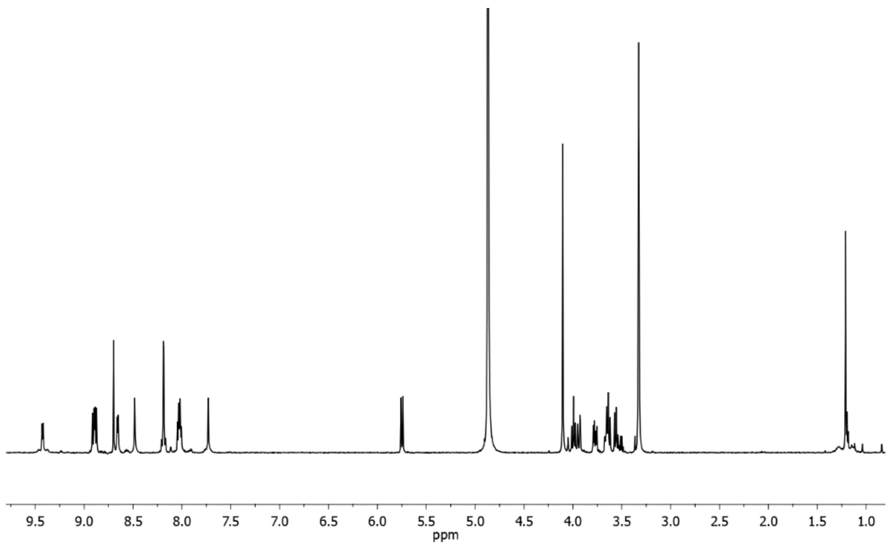

Figure S7. ^{1}H-NMR spectrum of **4Pt-im$_{\mathbf{dep}}$** in MeOD at 25 °C

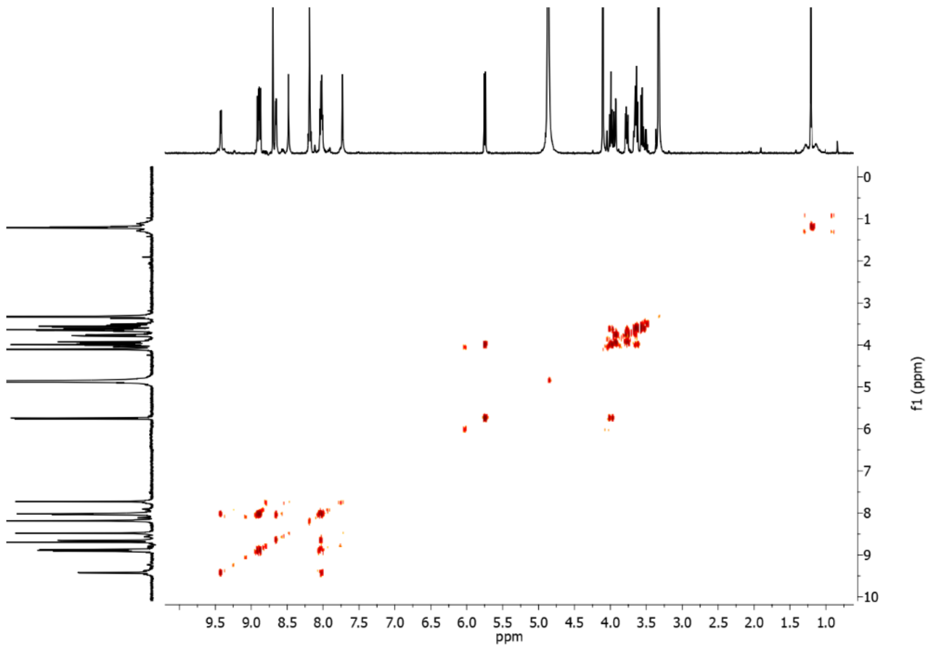

Figure S8. COSY NMR spectrum of 4Pt-im$_{dep}$ in MeOD at 25 °C

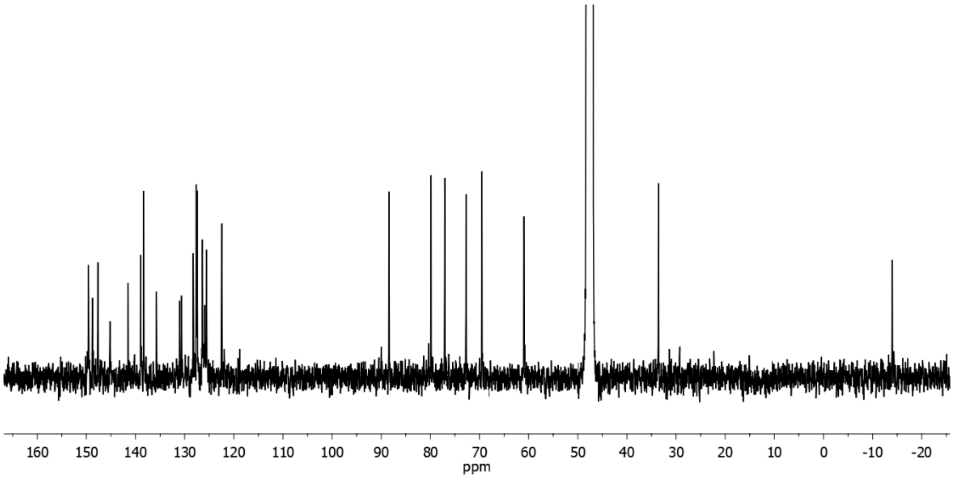

Figure S9. ^{13}C NMR spectrum of **4Pt-im$_{\mathbf{dep}}$** in MeOD at 25 °C

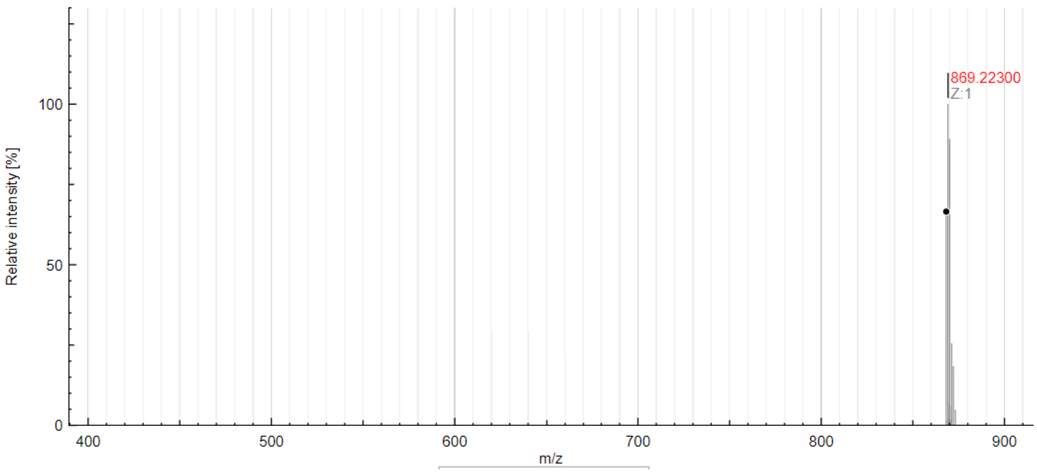

Figure S10. ESI-MS spectrum of **4Pt-im** in MeOH

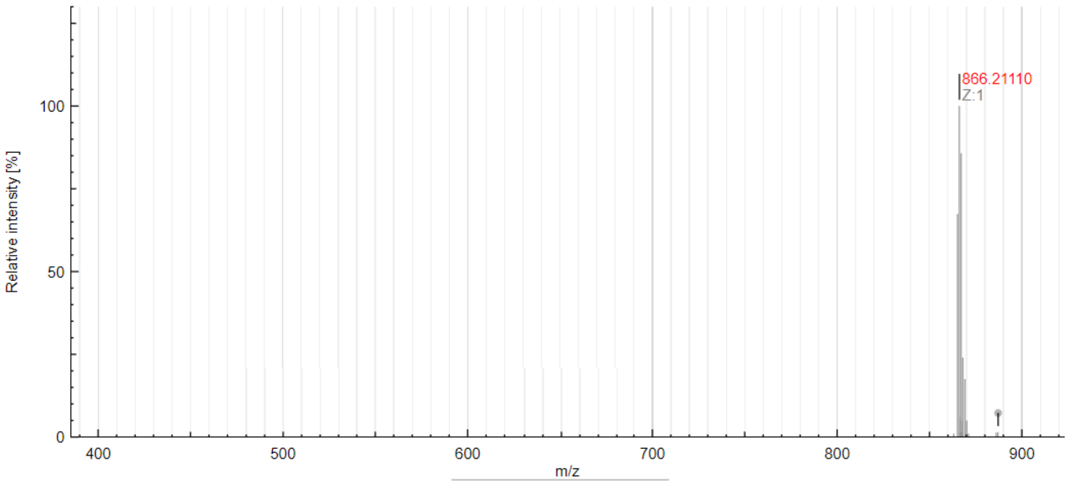

Figure S11 ESI-MS spectrum of **4Pt-py** in MeOH

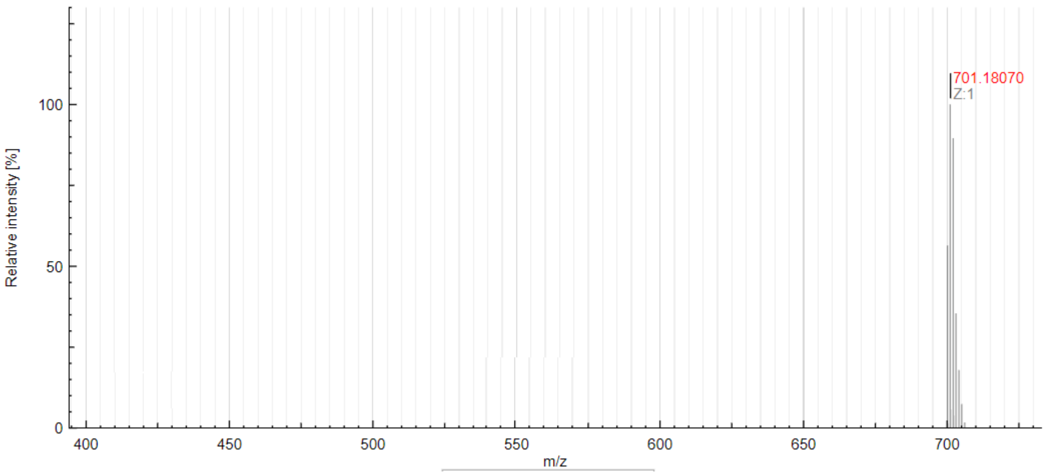

Figure S12 ESI-MS spectrum of **4Pt-im$_{dep}$** in MeOH

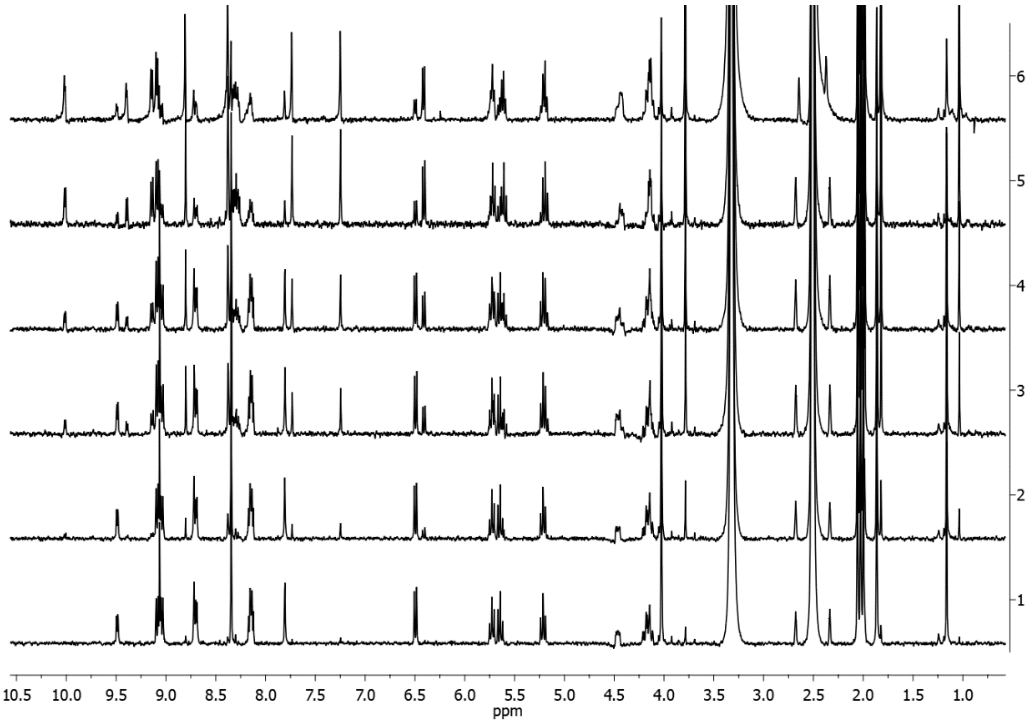

Figure S13. ^{1}H-NMR spectra over time of **4Pt-im** in DMSO (1 mM) at 37 °C (1 = start, 2 =1h, 3 = 3h, 4 = 5h, 5 = 24h, 6 =48 h).

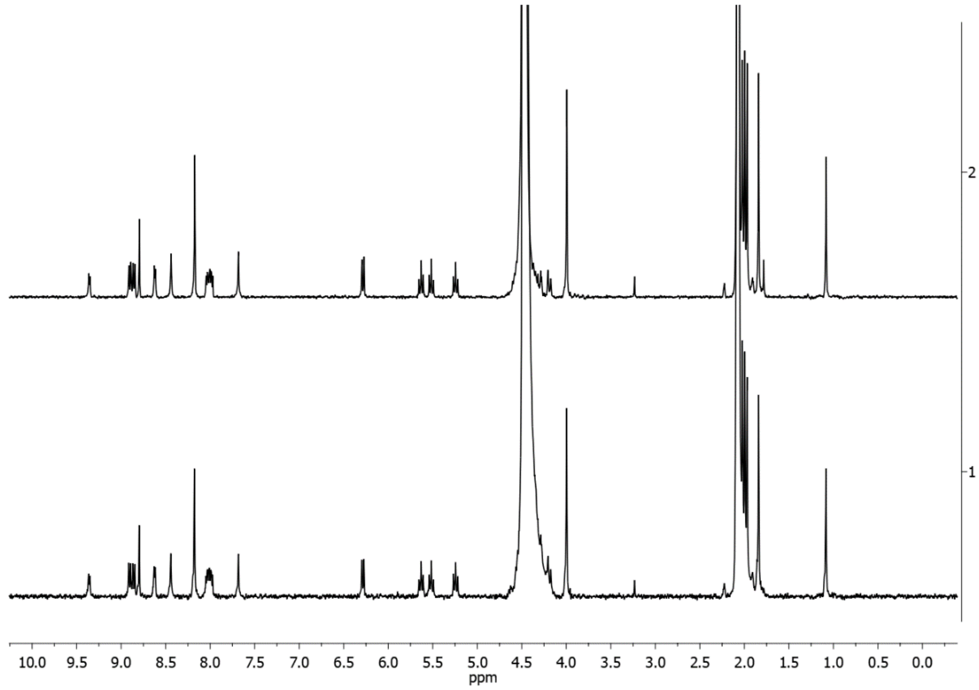

Figure S14. ^{1}H-NMR spectra over time of **4Pt-im** in 1:1 v/v D_2O : acetone-d_6 (1 mM) at 37 °C (1 = start, 2 =72 h).

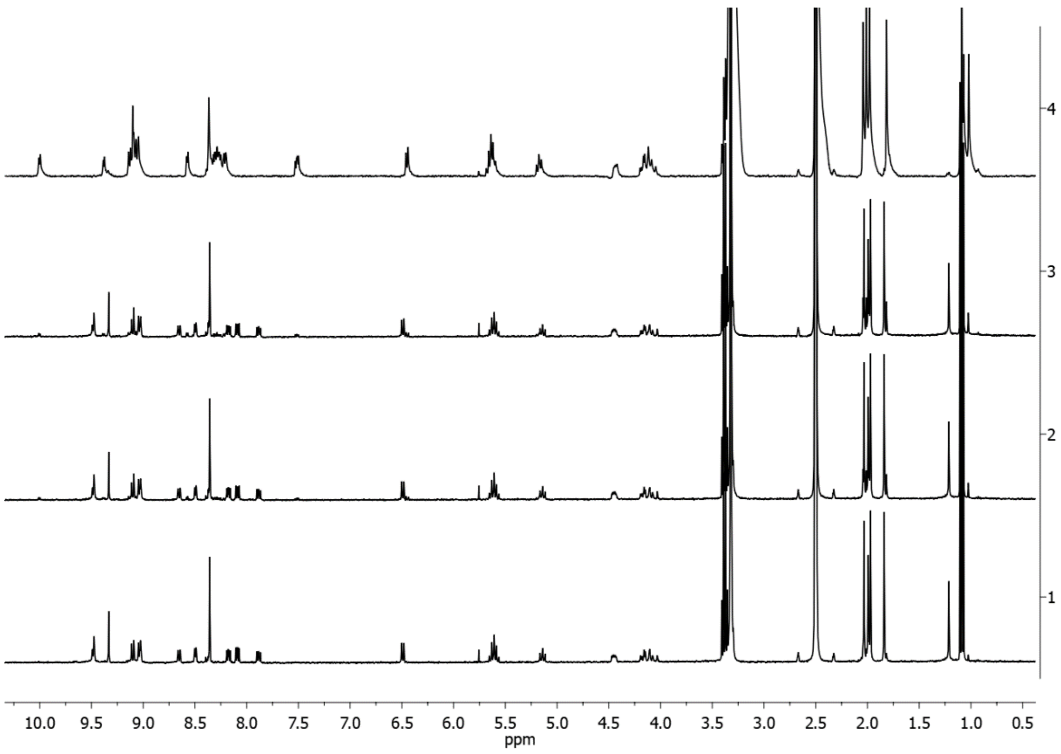

Figure S15. ^{1}H-NMR spectra over time of **4Pt-py** in DMSO (1 mM) at 37 °C (1 = start, 2 =1h, 3 = 2h, 4 = 48 h).

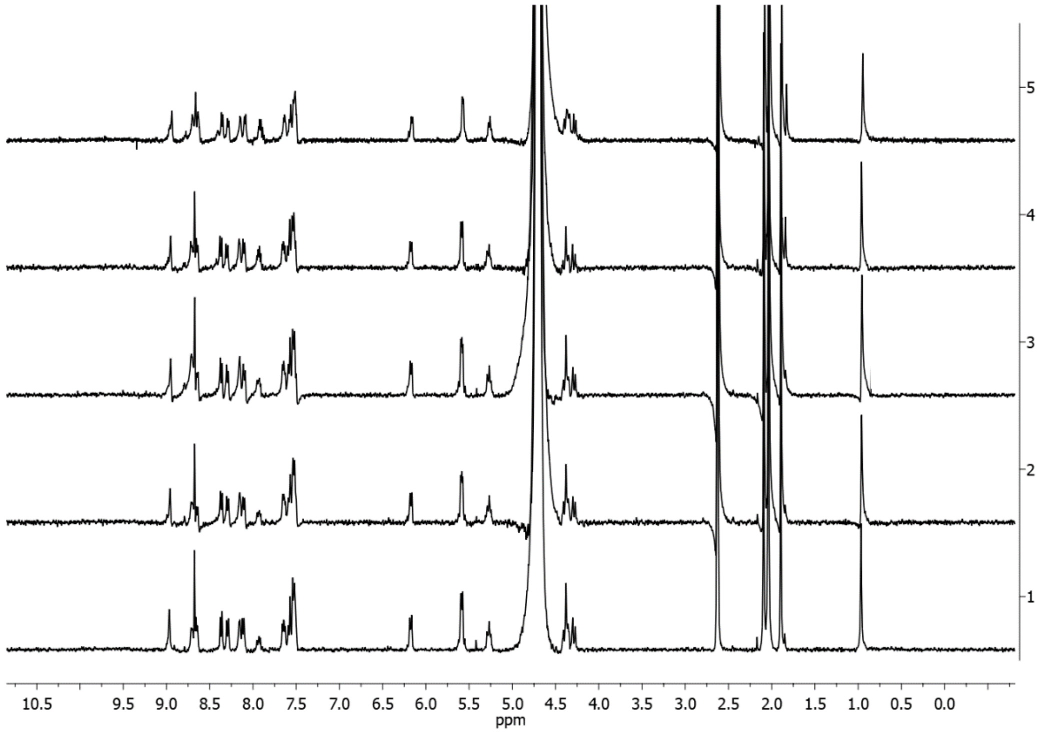

Figure S16. ^{1}H-NMR spectra over time of **4Pt-py** in 9:1 v/v PB : DMSO-d_6 (1 mM) at 37 °C (1 = start, 2 =1h, 3 = 3h, 4 = 24h, 5 =48 h).

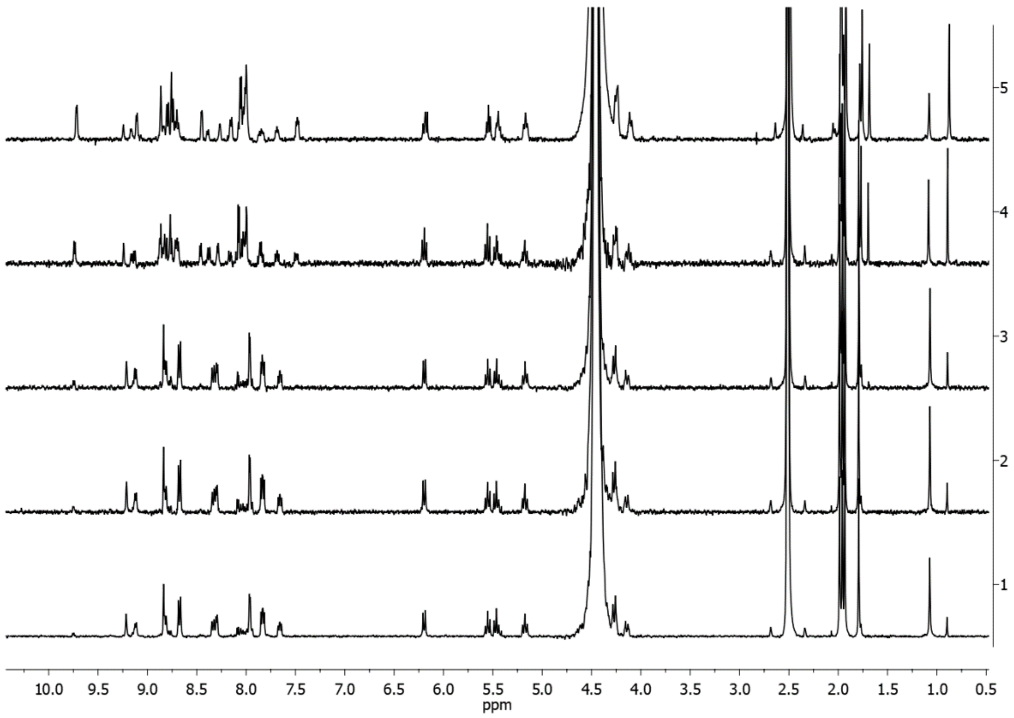

Figure S17. ^{1}H-NMR spectra over time of **4Pt-py** in 1:1 v/v PB : DMSO-d_6 (1 mM) at 37 °C (1 = start, 2 =1h, 3 = 3h, 4 = 24h, 5 =48 h).

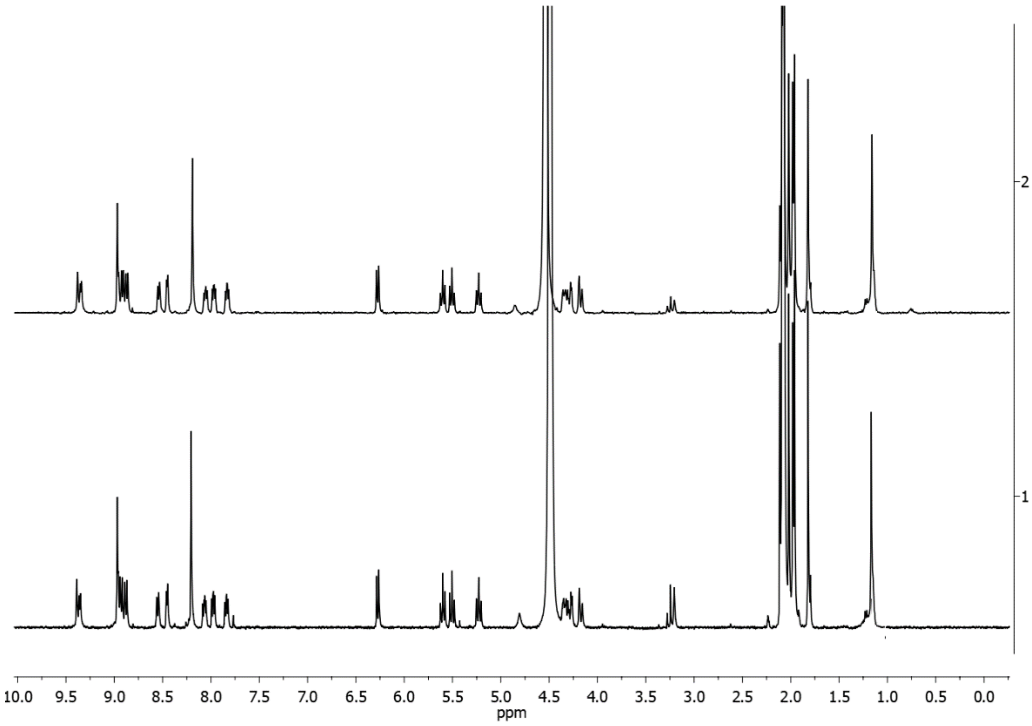

Figure S18. ^{1}H-NMR spectra over time of **4Pt-py** in 1:1 v/v D_2O : acetone-d_6 (1 mM) at 37 °C (1 = start, 2 =72 h).

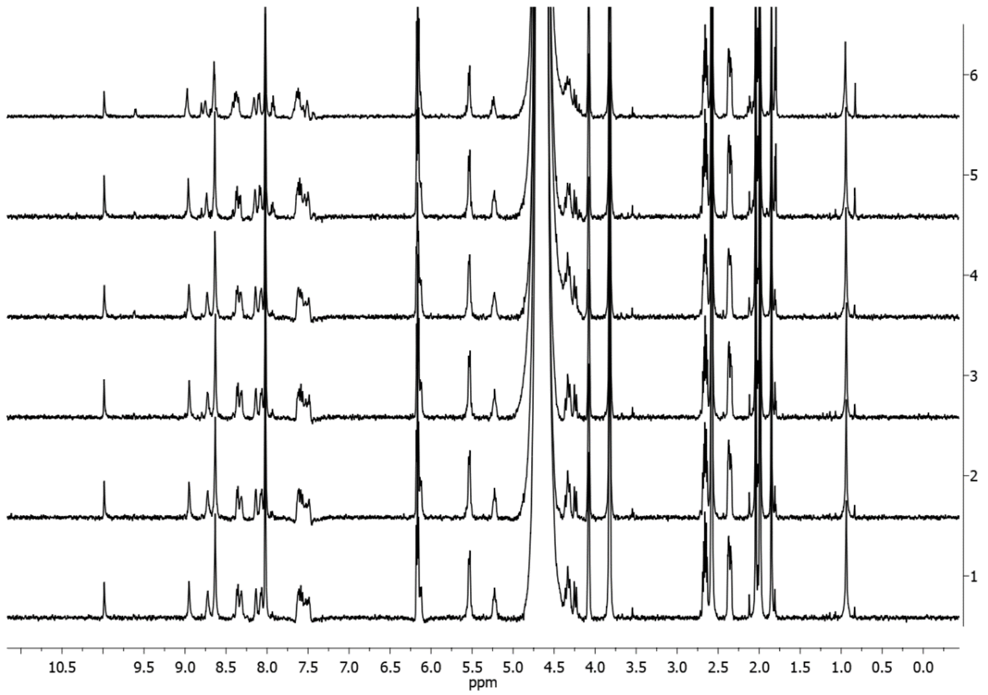

Figure S19. ^{1}H-NMR spectra of **4Pt-py** incubated with dGMP in 9:1 v/v PB:DMSO-d_6 (1 mM of Pt, 2.5 eq of dGMP) at 37 °C at different time (1 = start, 2 = 2h, 3 = 4h, 4 = 6h, 5 = 24 h, 6 = 48 h).

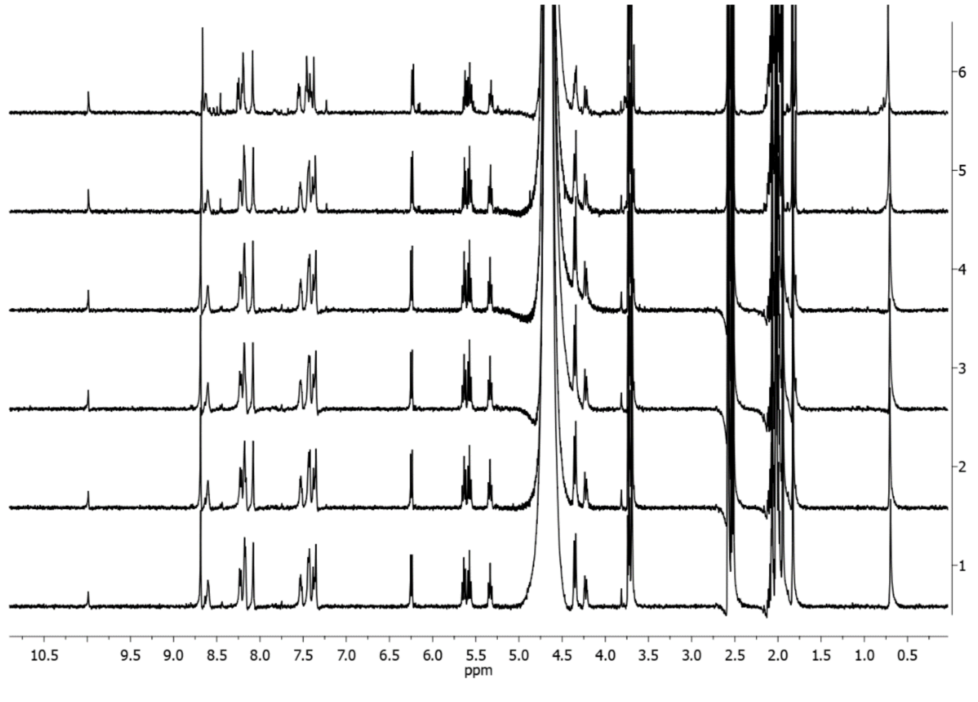

Figure S20. ^{1}H-NMR spectra of **4Pt-im** incubated with L-methionine in 9:1 v/v PB : DMSO-d_6 (1 mM of Pt, 2.5 eq of L-methionine) at 37 °C at different time (1 = start, 2 = 2h, 3 = 4h, 4 = 6h, 5 = 24 h, 6 = 48 h).

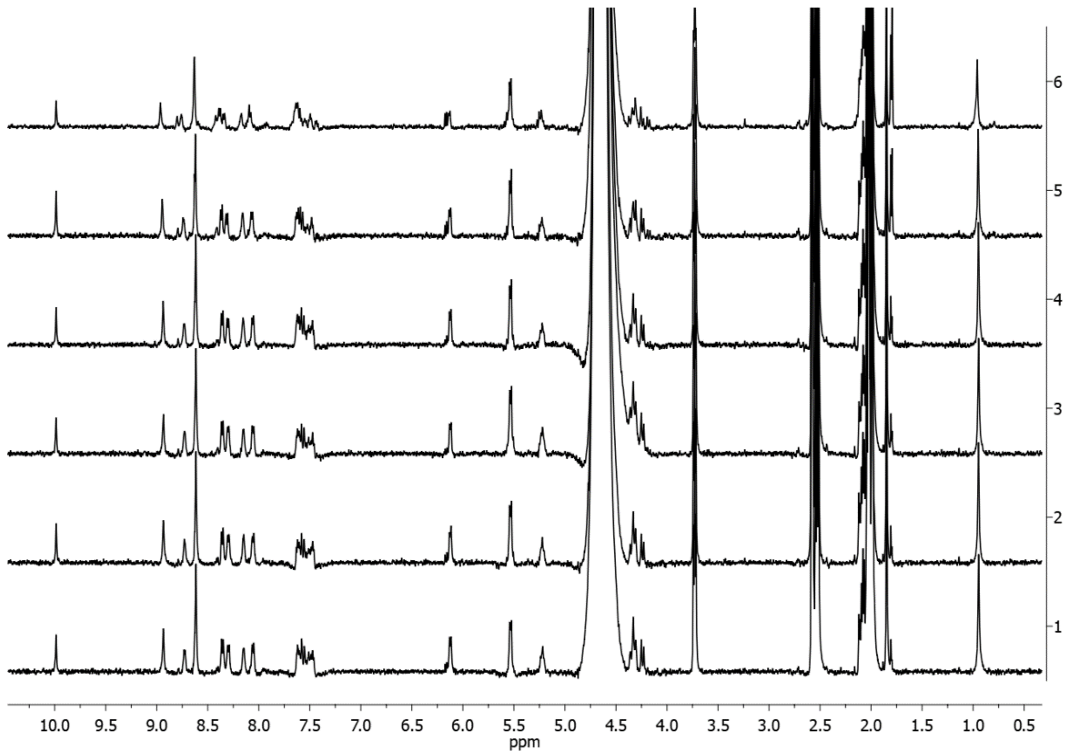

Figure S21. ^{1}H-NMR spectra of **4Pt-py** incubated with L-methionine in 9:1 v/v PB:DMSO-d_6 (1 mM of Pt, 2.5 eq of L-methionine) at 37 °C at different time (1 = start, 2 = 2h, 3 = 4h, 4 = 6h, 5 = 24 h, 6 = 48 h).

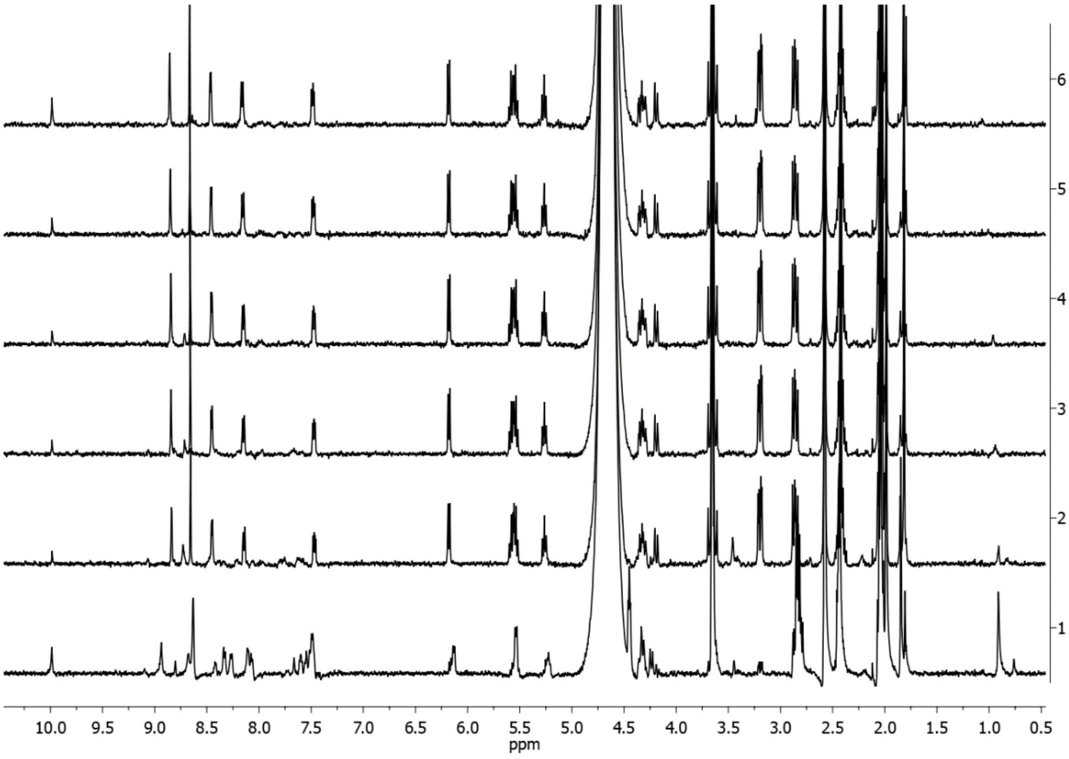

Figure S22. ^{1}H-NMR spectra of **4Pt-py** incubated with GSH in 9:1 v/v PB:DMSO-d_6 (1 mM of Pt, 2.5 eq of GSH) at 37 °C at different time (1 = start, 2 = 2h, 3 = 4h, 4 = 6h, 5 = 24 h, 6 = 48 h).

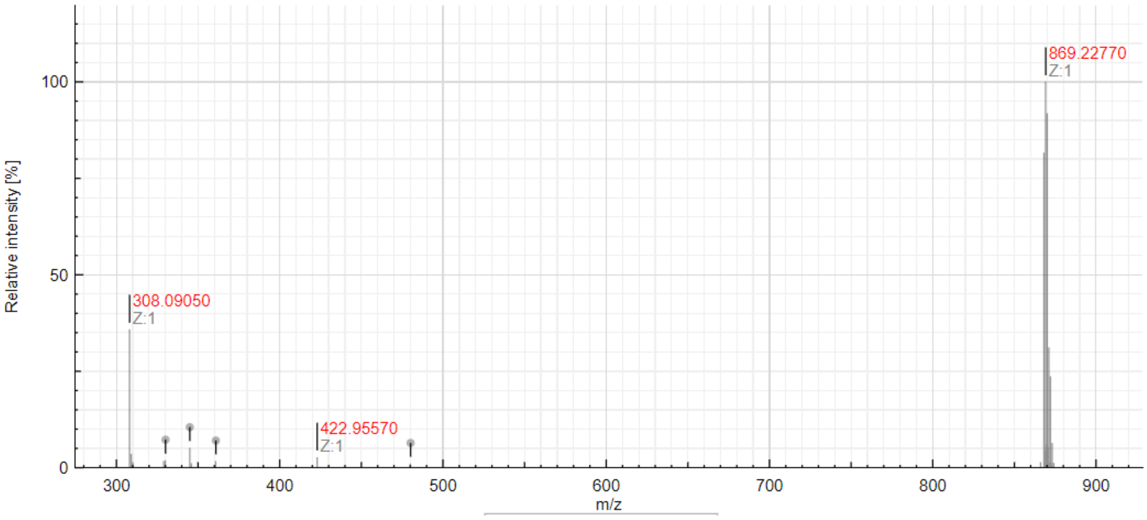

Figure S23. ESI-MS spectrum of **2Pt-im** incubated with GSH in 9:1 v/v H_2O : DMSO recorded after the dissolution.

Table S1. List of peaks found in the ESI-MS spectrum of the reaction mixture between **4Pt-im** and GSH (2.5 eq.) after 48 h of at 37 °C

Sequence	MF	Ionization	MF Mass	m/z	ppm	Intensity	Similarity
[PtPhenL2]	$C_{52}H_{58}N_{12}O_{18}Pt$	(+)2	1333.364	666.6814	1.355398	0.161499	0.987963
[4Pt-im]	$C_{33}H_{36}N_7O_9Pt$	(+)	869.2222	869.2217	1.418583	3.040044	0.980387
[Pt2Phen2GSH2-2H]	$C_{44}H_{48}N_{10}O_{12}Pt_2S_2$	(+)2	1362.219	681.109	1.774932	0.039036	0.968953
[PtMePhen]	$C_{13}H_{11}N_2Pt$	(+)	390.057	390.0565	-0.04174	0.087498	0.959729
[PtMePhenDmso]	$C_{15}H_{17}N_2OPtS$	(+)	468.071	468.0704	1.125112	2.147419	0.930783
[PtPhenGSH-H]	$C_{22}H_{23}N_5O_6PtS$	(H+)	680.1017	681.109	1.774932	0.039036	0.893688
[im + H]	$C_{20}H_{25}N_5O_9$	(H+)	479.1652	480.1725	1.405306	4.714523	0.951694
